# Multi-Target Neuroprotective Effects of Cordycepin and Adenosine from *Cordyceps militaris* Against Amyloid-β-Induced Neurotoxicity

**DOI:** 10.3390/biomedicines14081862

**Published:** 2026-08-20

**Authors:** Ewen Se Thoe, Hao Dong Tan, Ayesha Fauzi, Sunita Chamyuang, Yin Quan Tang, Adeline Yoke Yin Chia

**Affiliations:** 1 School of Biosciences, Faculty of Health and Medical Sciences, Taylor’s University, Subang Jaya 47500, Malaysiaayeshashahira.mfauzi@taylors.edu.my (A.F.); yinquan.tang@taylors.edu.my (Y.Q.T.); 2 Centre for Active Living, Taylor’s University, Subang Jaya 47500, Malaysia; 3 School of Science, Mae Fah Luang University, Chiang Rai 57100, Thailand; sunita@mfu.ac.th; 4 Microbial Products and Innovation Research Group, Mae Fah Luang University, Chiang Rai 57100, Thailand

**Keywords:** Alzheimer’s disease, β-secretase, Liver X receptors, *Cordyceps militaris*, nucleosides, drug discovery

## Abstract

**Background:** *Cordyceps militaris* (*C. militaris*) is a medicinal mushroom recognized for its diverse pharmacological activities, largely attributed to its principal bioactive nucleosides, cordycepin and adenosine. Although accumulating evidence supports their neuroprotective potential, the molecular mechanisms underlying their effects against Alzheimer’s disease (AD) remain incompletely understood. This study investigated the neuroprotective effects of cordycepin and adenosine against amyloid-β (Aβ_42_)-induced neurotoxicity and explored their potential molecular mechanisms using integrated experimental and computational approaches. **Methods:** SH-SY5Y neuroblastoma cells were pretreated with cordycepin (COR), adenosine (ADE), or donepezil (DNPZ) prior to Aβ_42_ exposure, and cell viability was assessed using the MTT assay. Drug-likeness and absorption, distribution, metabolism, excretion, and toxicity (ADMET) properties were evaluated in silico, followed by network pharmacology to identify potential therapeutic targets and enriched biological pathways. Molecular docking and molecular dynamics simulations were performed to elucidate the interactions of the compounds with selected Alzheimer’s disease-related proteins. **Results:** COR and ADE significantly attenuated Aβ_42_-induced cytotoxicity and improved SH-SY5Y cell viability. Network pharmacology identified 84 shared molecular targets, including 9 AD-associated genes. Protein–protein interaction analysis revealed hub genes involved in signal transduction, epigenetic regulation, and purine metabolism, while Gene Ontology and KEGG enrichment analyses highlighted pathways associated with neuroactive ligand–receptor interaction, calcium signaling, and inflammatory regulation. ADMET analysis predicted favorable pharmacokinetic properties for both compounds, although cordycepin was predicted to be AMES-positive. Molecular docking and molecular dynamics simulations demonstrated stable interactions of COR and ADE with liver X receptors (LXRα and LXRβ), whereas donepezil exhibited stronger binding affinity toward β-secretase (BACE1). **Conclusions:** COR and ADE exert neuroprotective effects through coordinated modulation of multiple AD-related signaling pathways rather than a single molecular target. These findings provide mechanistic insights into the neuroprotective activities of *C. militaris*-derived nucleosides and support further investigation of their potential as multi-target therapeutic candidates for AD and other neurodegenerative disorders.

## 1. Introduction

Alzheimer’s disease (AD) is a neurological-based degenerative disease that primarily targets the elderly generation by promoting cognitive and memory impairment [1]. According to the reports generated by World Health Organization (WHO), the prevalence of AD is projected to hit approximately 152 million people worldwide by year 2050, considering that we are slowly moving towards an aging society [2]. From a pathological perspective, AD is clinically characterized by the abnormal accumulation of extracellular amyloid-β (Aβ) plaques and intracellular neurofibrillary tangles (NFT) [3,4]. AD is categorized into familial AD (FAD), early-onset AD (EOAD) and late-onset AD (LOAD), the former two accounting to <5% while the latter consisted of >95% of the affected population [5]. Mounting evidence suggested mutations in several causative genes such as amyloid precursor protein (APP), presenilin 1 (PSEN1) and presenilin 2 (PSEN2) responsible for EOAD or FAD emergence [6]. Notably, processing of amyloid precursor protein (APP) by β-secretase (BACE1) at the Asp site of Aβ domain generates soluble ectodomain fragment (sAPPβ) and membrane-bound C-terminal fragment (C99) [7,8,9]. Further cleavage by β-secretase then stimulates production of APP intracellular domain (AICD) and plaque-inducing Aβ_40_ or Aβ_42_ peptides [10]. In light of the pathogenic role of BACE1, this enzyme has been recognized as a potential inhibitory target for improving cognitive deficit in AD [11].

On the contrary, apolipoprotein E (APOE) was revealed as a susceptibility gene leading to LOAD development; with the ε4 allele presenting as the strongest risk component as shown in genome-wide association studies (GWAS) [12,13]. Many studies have reported that APOE transcription is driven by nuclear receptors, such as Retinoid X Receptors (RXRs) and liver X receptors (LXRs), which couple up to form permissive heterodimers [14]. The expression of two different isoforms of LXR (α, β) are critical in modulating lipid homeostasis, inflammatory responses and cell proliferation [15,16,17]. Activation of LXRs has been shown to promote APOE lipidation by mediating the activity of ATP-binding cassette A1 (ABCA1), which in turn facilitates cholesterol and phospholipid efflux onto extracellular APOE to induce pre-HDL-like particles [18,19,20]. Formation of mature HDL-like particles over time indicates successful APOE lipidation, thus reducing neurotoxic Aβ secretion [21].

To date, oral administration of approved pharmaceutical drugs remains the conventional method for the majority of the public in an attempt to halt AD progression [22,23]. While a certain level of neuroprotection was indeed induced, they were ultimately noted to provide symptomatic relief without eliminating the root cause [24]. Accompanied by adverse side effects, it is also important to pinpoint that these drugs were initially designed to target acetylcholinesterase receptors (e.g., donepezil, rivastigmine, galantamine) or glutamate receptors (e.g., memantine). Therefore, it is likely that the above drugs might not be able to work effectively against other AD-related signaling pathways. The failure of standard regimes for AD treatment underscores the demand to develop novel and specific neuroprotective agents.

Historically, medicinal mushrooms appear to be one of the most dominant sources of natural products in drug discovery. *Cordyceps millitaris* (*C. militaris*), a medicinal fungi of the Clavicipitaceae family, had received immense attention due to its well-cultivated nature under laboratory conditions [25,26]. Among medicinal mushrooms, *C. militaris* has emerged as one of the most extensively studied species due to its long history of use in traditional medicine and its ability to be cultivated under controlled laboratory conditions. Numerous pharmacological studies have reported antioxidant, anti-inflammatory, immunomodulatory, anti-tumor and neuroprotective activities associated with *C. militaris* extracts and purified constituents. These biological properties have attracted considerable interest in the development of natural product-based therapeutics for chronic diseases, particularly those involving oxidative stress, inflammation and age-related tissue degeneration. Given that neuroinflammation, oxidative damage and metabolic dysregulation are recognized contributors to Alzheimer’s disease pathogenesis, bioactive compounds derived from *C. militaris* represent promising candidates for further investigation as multi-target neuroprotective agents.

The therapeutic potential of *C. militaris* is largely attributed to its diverse repertoire of bioactive metabolites, including polysaccharides, sterols, peptides and nucleoside derivatives. Therapeutic potential exhibited by *C. militaris* is believed to highly correspond with its chemical constituents, in which the nucleoside analogs cordycepin (3′-deoxyadenosine) and adenosine are among the most commonly investigated compounds [27,28,29,30]. Cordycepin (COR) and adenosine (ADE) are regarded as the principal pharmacologically active nucleosides and have been widely investigated for their anti-inflammatory, antioxidant, immunomodulatory and neuroprotective properties. Although the therapeutic effects of *C. militaris*-derived COR and ADE have been explored across multiple in vitro and in silico studies, there are so far no reports that delved into the binding mechanism of both nucleoside analogs towards target protein BACE1 and LXRs (-α, -β). The molecular mechanisms through which cordycepin and adenosine modulate AD-related targets remain incompletely understood.

**Figure 1:**
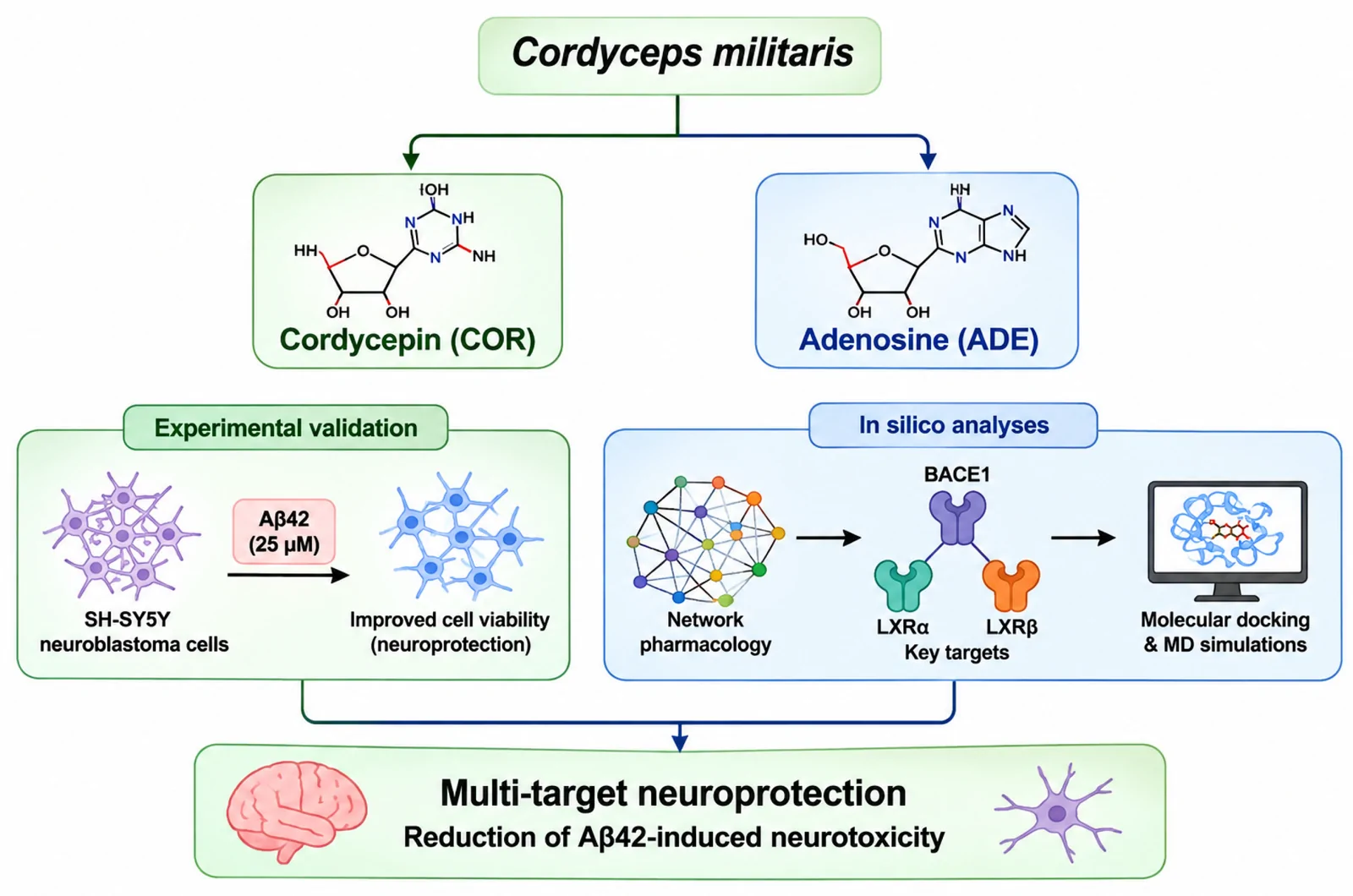
Schematic depiction of the experimental workflow and postulated mechanisms of the neuroprotective effects of cordycepin (COR) and adenosine (ADE) from *Cordyceps militaris*. In this study, combined in vitro validation in Aβ_42_-induced SH-SY5Y neuroblastoma cells and in silico analyses, such as network pharmacology, molecular docking, and molecular dynamics (MD) simulations. Structure-based analyses were performed on BACE1, LXRα and LXRβ as representative therapeutic targets related to Alzheimer’s disease. The integrated workflow illustrates the proposed multi-target actions of COR and ADE in reducing the neurotoxicity of Aβ_42_.

Hence, this study aimed to investigate the neuroprotective effects of the two major bioactive nucleosides of *C. militaris*, COR and ADE, against Aβ_42_-induced neurotoxicity using an integrated experimental and computational framework. Network pharmacology was employed to identify potential molecular targets and biological pathways, while molecular docking and molecular dynamics (MD) simulations were performed against BACE1, LXRα and LXRβ, key regulators of amyloidogenesis and APOE-mediated lipid metabolism implicated in AD. A summary of the image of study is illustrated in Figure 1.

## 2. Materials and Methods

### 2.1. Preparation of Cordycepin and Adenosine

*C. militaris* was kindly provided by Dr. Sunita Chamyuang (Mae Fah Luang University, Chiang Rai, Thailand). The fungal culture originated from the laboratory collection of Dr. Chamyuang throughout the study. All details regarding the cultivation parameters were considered private and confidential. As such, the isolate has not been deposited in any publicly accessible culture collection.

COR and ADE utilized in this study were isolated from the above fungal strain. Briefly, freeze-dried fruiting body and mycelial powder of *C. militaris* were subjected to aqueous and acetonitrile extraction. The extracts were filtered prior to High-Performance Liquid Chromatography (HPLC), (Shimadzu, Kyoto, Japan) coupled with Quadrupole Time-of-Flight Liquid Chromatography Tandem Mass Spectrometry (QTOF LC/MS-MS) (Agilent Technologies Inc., Santa Clara, CA, USA) analysis for purification and identification of the compounds COR and ADE. The compound identity was validated using standard COR (Identifier No. C3394) and ADE (Identifier No. A9251) directly purchased from Merck (Darmstadt, Germany). COR and ADE purified to ≥95% were freeze-dried and stored at −20 °C until further use. The compounds were dissolved in sterile distilled water for generation of stock solutions, and freshly diluted to required working concentrations using complete culture medium prior to cellular treatment.

### 2.2. Cell Culture

Human neuroblastoma SH-SY5Y cell line was purchased from iCell Bioscience Inc. (Shanghai, China) and cultured in a 1:1 mixture of MEM and F12 Medium, supplemented with 1% Glutamax, 1% sodium pyruvate, 1% Non-Essential Amino Acid (NEAA), 15% Fetal Bovine Serum (FBS) and 1% penicillin/streptomycin solution (Nacalai Tesque, Kyoto, Japan). The cells were incubated in a humidified atmosphere of 95% air and 5% CO_2_ at 37 °C (Eppendorf, Hamburg, Germany).

### 2.3. Cell Viability Assay

Cell viability assay was performed on undifferentiated SH-SY5Y neuroblastoma cells using the 3-(4,5-dimethylhiazol-2-yl)-2,5-diphenyltetrazolium bromide (MTT) method [31]. SH-SY5Y cells were seeded into 96-well plates at a density of 4 × 10^4^ per well and incubated overnight. After the formation of a monolayer of cells, the cells were pretreated with COR, ADE or standard drug donepezil (DNPZ) for 24 h. Aβ_42_ fibrils were then added into the cells and subjected to another 24 h incubation. A total of 20 μL of MTT solution (5 mg/mL) was aspirated into each well and incubated for 3 and a half hours in the dark. Next, all media were discarded and replaced with 150 μL dimethyl sulfoxide (DMSO) to dissolve the purple formazan crystals. Absorbance readings were measured at 570 nm using a microplate spectrophotometer to evaluate the neuroprotective effects of respective treatment groups against Aβ_42_ fibril-induced cytotoxicity.

To measure the maximum non-toxic concentration (MNTC) of COR, ADE and DNPZ on SH-SY5Y cells, 100 μL of various concentrations of COR, ADE (7.81–500.00 μg/mL) or DNPZ (0.42–16.64 μg/mL) were added into the seeded cells for 24 h. Moving on, the MTT procedure was repeated and EC_90_ values indicating the lowest cytotoxicity of COR, ADE and DNPZ in preserving >90% cell viability percentage was determined. The EC_90_ values were recognized as MNTC and selected for further experiments. For the assessment of Aβ_42_ fibrillar toxicity, the seeded cells were incubated with Aβ_42_ fibrils ranging from 0 to 30 μM for 24 h. Prior to Aβ_42_ fibrils addition, some of the cell culture medium was carefully removed from the wells without disturbing the cell layer to adjust to the desired final concentrations of Aβ_42_ fibrils. MTT procedure was similarly carried out and the optimal concentration that decreases cell viability is chosen for further assays.

### 2.4. Physicochemical and Pharmacokinetic Analysis

The drug-likeliness properties and molecular descriptors of COR, ADE and DNPZ were predicted with the Molinspiration server (http://www.molinspiration.com; accessed 1 November 2025) [32]. Lipinski Rule of Five was utilized to examine the oral bioavailability of the pure compounds and standard drug [33]. Moreover, admetSAR (https://lmmd.ecust.edu.cn/admetsar2/; accessed 1 November 2025) was used to screen their chemical ADMET profiles (Absorption, Distribution, Metabolism, Excretion and Toxicity) [34]. To further evaluate the predicted mutagenicity profiles obtained from admetSAR, cordycepin and adenosine were independently analyzed using the ProTox-3.0 web server (https://tox.charite.de/protox3; accessed 1 August 2026). The canonical SMILES of each compound were used as input, and toxicity endpoints including carcinogenicity, mutagenicity, and cytotoxicity were evaluated using the default prediction models. The resulting predictions were compared with the admetSAR toxicity profiles to assess the consistency of the computational toxicity assessment.

### 2.5. Network Pharmacology Analysis

#### 2.5.1. Identification of Compound-Related Targets

Potential molecular targets of COR and ADE were identified using the SwissTargetPrediction database (http://www.swisstargetprediction.ch/; accessed 1 November 2025). The canonical SMILES structures of COR (C1[C@H](O[C@H]([C@@H]1O)N2C=NC3=C(N=CN=C32)N)CO) and ADE (C1=NC(=C2C(=N1)N(C=N2)[C@H]3[C@@H]([C@@H]([C@H](O3)CO)O)O)N) were retrieved from the PubChem database and used as query inputs. The species was restricted to Homo sapiens, and all predicted targets were collected after removal of duplicate entries.

#### 2.5.2. Identification of Alzheimer’s Disease-Associated Genes

AD-related genes were retrieved from the GeneCards database (https://www.genecards.org/; accessed 1 November 2025) using the keyword “Alzheimer’s disease”. To improve disease specificity and reduce background noise, genes were ranked according to their GeneCards relevance scores, and the top 500 protein-coding genes were selected for further analysis.

#### 2.5.3. Construction of Protein–Protein Network

The predicted targets of COR and ADE, together with AD-associated genes, were imported into Venny 2.1 (https://bioinfogp.cnb.csic.es/tools/venny/; accessed 1 November 2025) to identify overlapping targets. A total of 84 shared targets between COR and ADE were identified. These 84 shared targets were subsequently used for protein–protein interaction (PPI) network construction and functional enrichment analyses, including Gene Ontology (GO) and Kyoto Encyclopedia of Genes and Genomes (KEGG) pathway analyses. Separately, the 84 shared targets were intersected with AD-associated genes to identify nine core disease-related targets for subsequent biological interpretation. Venn diagrams were generated to visualize the overlap among the datasets.

The 84 shared targets were imported into the STRING database (https://string-db.org/; accessed 1 November 2025) for protein–protein interaction (PPI) analysis. The organism was restricted to Homo sapiens, and interactions with a confidence score > 0.4 were retained. The resulting interaction network was exported and visualized using Cytoscape software (version 3.10.4). Topological analysis of the PPI network was performed using the NetworkAnalyzer tool in Cytoscape. Network parameters, including degree centrality, betweenness centrality, and neighborhood connectivity, were calculated. Genes with the highest degree values were identified as hub genes and selected for subsequent biological interpretation as potential key regulators of the neuroprotective activity of COR and ADE.

#### 2.5.4. Gene Ontology and KEGG Pathway Enrichment Analysis

Functional enrichment analyses were performed using Metascape (https://metascape.org/gp/index.html#/main/step1; accessed 1 November 2025) and Enrichr (https://maayanlab.cloud/Enrichr/; accessed 1 November 2025) platforms. The 84 shared targets identified between COR and ADE were used for Gene Ontology (GO) and Kyoto Encyclopedia of Genes and Genomes (KEGG) pathway enrichment analyses. GO enrichment analysis was conducted to investigate Biological Processes (BP), Molecular Functions (MF), and Cellular Components (CC) associated with the shared targets. KEGG pathway enrichment analysis was subsequently performed to identify significantly enriched signaling pathways. Enriched terms with a *p*-value < 0.05 were considered statistically significant and ranked according to enrichment significance.

### 2.6. Ligand Preparation

The chemical structures of cordycepin (PubChem CID: 6303), adenosine (PubChem CID: 60961) and donepezil (PubChem CID: 5741) were downloaded from PubChem in SDF format (https://pubchem.ncbi.nlm.nih.gov; accessed 1 December 2025) [35]. DNPZ was included as a reference ligand for comparative docking analysis. Polar hydrogen atoms and Gasteiger partial atomic charges were applied to the ligands using AutoDockTools v.1.5.7. The number of atoms in each ligand were also checked before individually saving and converting them in PDBQT format.

### 2.7. Protein Receptor Preparation

BACE1 and liver X receptors (LXRα and LXRβ) were selected as representative therapeutic targets based on their well-established roles in Alzheimer’s disease pathology. BACE1 is a key enzyme involved in amyloid precursor protein processing and amyloid-β generation, whereas LXRα and LXRβ regulate APOE-mediated lipid metabolism and amyloid-β clearance. Although these receptors were not identified among the overlapping disease-associated targets in the network pharmacology analysis, they were selected through a literature-guided approach because they represent canonical Alzheimer’s disease targets with experimentally resolved crystal structures suitable for structure-based molecular modeling. The 3D crystallographic structures of BACE1 (6JSE, resolution = 2.00 Å), LXR-α (3IPQ, resolution = 2.00 Å) and LXR-β (6S5K, resolution = 1.60 Å) were retrieved from RCSB Protein Data Bank (PDB) (http://www.rcsb.org; accessed 1 December 2025). The protein receptors were edited through AutoDockTools v.1.5.7. accordingly: (i) removal of bound ligands, (ii) deletion of water molecules, (iii) addition of polar hydrogen atoms, (iv) incorporation of missing side chains, (v) assignment of Kollman and Gasteiger partial atomic charges. The protein receptor files were saved and converted to PDBQT format for later use.

### 2.8. Determination of Binding Pockets in Target Protein

Each protein receptor was submitted to the online CASTp server (Computed Atlas of Surface Topography of Proteins) v.3.0 (http://cast.engr.uic.edu; accessed 1 December 2025) for detection of potential binding pockets [36]. The first pocket showing the largest volume and surface area was identified as most favorable for ligand binding. Prior to docking, the grid box was designated using AutoGrid4 in a way that encompasses all the amino acids present within the predicted protein pocket. The center for the grid box was positioned at (x: 17.914, y: 41.592, z: −5.513) with 80 × 80 × 70 grid map for BACE1. As for LXR-α, the coordinates and number of dimension points was set at (x: 42.185, y: 22.199, z: −2.542) and (x: 100, y: 90, z: 70); while for LXR-β it was (x: −11.548, y: −11.743, z: −16.879) and (x: 80, y: 70, z: 60), respectively. All parameters were saved as grid parameter files before starting the docking run. The dimensions and positions of the docking grid boxes were visually verified to ensure complete coverage of the entire CASTp-predicted binding pocket and all constituent pocket residues for each receptor. Representative overlays of the grid boxes on the predicted binding pockets are provided in Supplementary Figure S1.

### 2.9. Molecular Docking

To explore the binding association between desired compounds and selected protein targets, a molecular docking study was conducted using AutoDock Vina (Version 1.2.5) [37]. Each ligand–protein complex was subjected to 100 docking runs and the resulting output files containing respective binding energies (kcal/mol) are reported in text data format. The intermolecular interactions and binding pose of the models were analyzed and visualized using BIOVIA Discovery Studio Visualizer 2021 (Dassault Systèmes BIOVIA, San Diego, CA, USA). The docking protocol was validated by re-docking the co-crystallized ligand into the binding site using the same docking parameters employed for the test compounds. The reproduced binding pose confirmed that the selected docking protocol was able to recover the experimental binding mode and was therefore considered suitable for subsequent docking analyses.

### 2.10. Molecular Dynamics Simulation

Based on docking results, only docked complexes portraying the best pose and lowest energy values were selected for MD simulations using the CHARMM36 all-atom force field available in Groningen Machine for Chemicals Simulations GROMACS-2019 software packages (GNU, General Public License; http://www.gromacs.org; first accessed 1 December 2025) [38]. The ligand topology files were generated using CHARMM General Force Field (CGenFF) server (ParamChem project; https://cgenff.com/; first accessed 1 December 2025), while protein topologies were assigned using the CHARMM36 force fields [39]. Each ligand–protein complex was solvated in a dodecahedral periodic box containing TIP3P water models extending at least 10 Å from the protein surface and neutralized with sodium (Na^+^) and chloride (Cl^−^) ions.

Energy minimization was performed using the steepest descent algorithm (nsteps = 50,000) for a period of 100,000 iterations (100 ps), followed by a two-step equilibrium through NVT and NPT ensemble to eliminate any undesirable contacts. During the process of thermalization, a temperature of 300 K was maintained and the pressure kept at 1 bar. Particle Mesh Ewald (PME) algorithm was computed for long-range electrostatic forces, and the cut-off radius applied to Couloumb and van der Waals interactions were fixed at 1.2 nm; while all hydrogen bonds constrained through Linear Constraint Solver (LINCS).

MD production was subsequently performed for 50 ns. Structural stability was evaluated using root mean square deviation (RMSD), root mean square fluctuation (RMSF), and radius of gyration (Rg) calculated with the built-in GROMACS analysis tools, while plots were generated using XmGrace (Version 5. 1.25). Intermolecular hydrogen bond occupancy was analyzed using the Hydrogen Bond plugin in Visual Molecular Dynamics (VMD) (Version 1.9.4) (the University of Illinois at Urbana-Champaign, USA) [40].

### 2.11. Binding Free Energy Calculation by MMGBSA Analysis

To quantitatively estimate the binding affinity of protein–ligand complexes, Molecular Mechanics/Generalized Born Surface Area (MM/GBSA) analysis was performed using MMPBSA v1.6.3 based on the MMPBSA.py v16.0 script [41,42]. Trajectory snapshots were extracted at regular intervals of every 100 ps from the MD production simulations, yielding 500 snapshots for calculation of binding free energy. The binding free energy (ΔG_bind) measures how strongly the ligand will bind to its target (protein or receptor), and the stability of interaction between two molecules.

### 2.12. Statistical Analysis

Three independent experiments with three replicates were carried out. All data were presented as mean ± standard deviation (SD). Statistical analyses were conducted using one-way analysis of variance (ANOVA), followed by Tukey’s multiple comparison test with GraphPad Prism version 8.3.1 (GraphPad Software Inc., San Diego, CA, USA). A *p*-value less than 0.05 or 0.01 indicate significant difference between the groups.

## 3. Results

### 3.1. In Vitro Cell Viability Screening

#### 3.1.1. Maximum Non-Toxic Concentration of COR, ADE and DNPZ on SH-SY5Y Cells

**Figure 2:**
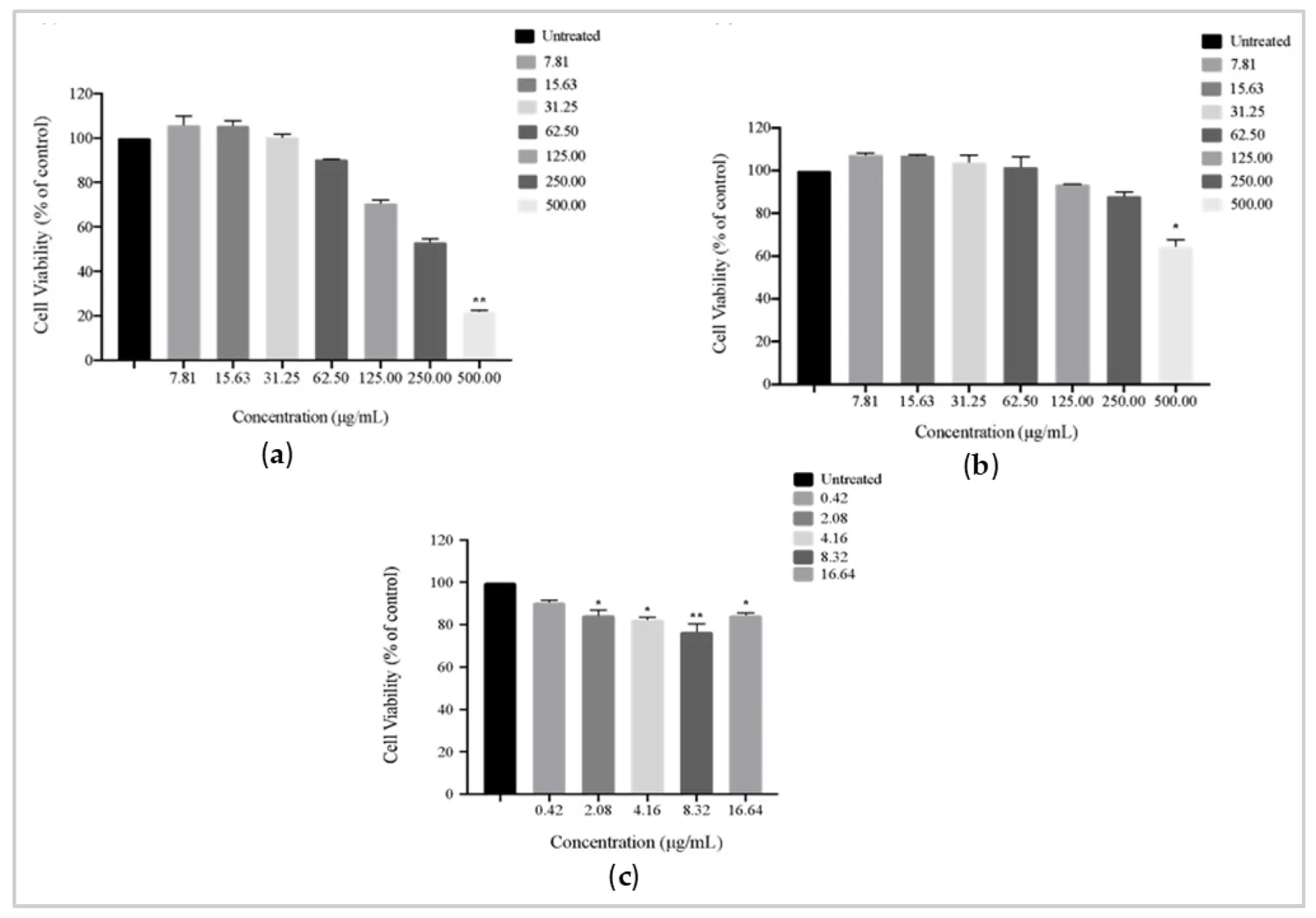
Cell viability effects of SH-SY5Y cells following incubation with various concentration gradients of (**a**) COR (7.81–500.00 μg/mL), (**b**) ADE (7.81–500.00 μg/mL) and (**c**) DNPZ (0.42–16.64 μg/mL) at 24 h. Data were expressed as mean ± SD from three independent experiments performed in triplicates. Statistically significant difference vs. untreated group: * *p* < 0.05, ** *p* < 0.01.

COR and ADE (7.81–500.00 μg/mL), as well as DNPZ (0.42–16.64 μg/mL), were utilized to treat SH-SY5Y cells for 24 h to investigate whether they induce cytotoxicity to the cells, and the results are demonstrated in Figure 2. Undifferentiated SH-SY5Y cells are used as an AD cellular model in this study. The MNTC of COR, ADE and DNPZ was determined and expressed by EC_90_ values, based on the prospect that cell viability remained intact (above 90%) following test compound and standard drug treatment. In general, natural product-derived medicines possess innate toxic elements that are yet to be fully distinguished from therapeutic components [43]. The presence of these harmful phytochemicals alongside heavy metals that are not properly filtered can potentially damage our body system, in some cases even promoting neuronal impairment [44,45,46,47]. Hence, it is paramount to conduct toxicity screening of an intended natural product target before mass production [48].

Interestingly, incubation of SH-SY5Y cells with COR and ADE compounds resulted in a decrease in cell viability in a dose-dependent manner. Of note, treatment with COR and ADE at the highest concentration 500 μg/mL significantly reduced cell viability up to 22.07 ± 0.53 and 64.58 ± 2.92, respectively (Figure 2a,b). On the other hand, DNPZ demonstrated a concentration-dependent cellular survival ranging from 0.42 to 8.32 μg/mL, depicting a steady drop in cell viability that switched to sudden spike at 16.64 μg/mL instead. The significant decline in cell viability is revealed as 84.59 ± 2.30 at 2.08 μg/mL, 82.63 ± 0.88 at 4.16 μg/mL, 76.85 ± 3.67 at 8.32 μg/mL and 84.73 ± 1.04 at 16.64 μg/mL (Figure 2c). With this, we observed the EC_90_ of COR to fall within the concentration range of 7.81–62.50 μg/mL; while a higher concentration ranging between 7.81 μg/mL and 125.00 μg/mL was reported for ADE. Meanwhile, DNPZ serving as the positive control achieved EC_90_ at an individual concentration of 0.42 μg/mL. The lowest concentration among the selected EC_90_ values in respect to different treatment groups was then chosen as MNTC. As such, the MNTC for COR and ADE is set to be 7.81 μg/mL, whereas for DNPZ it is 0.42 μg/mL as almost no toxicity was registered at that particular concentration.

#### 3.1.2. Cytotoxicity of Aβ_42_ Fibrils on SH-SY5Y Cells

**Figure 3:**
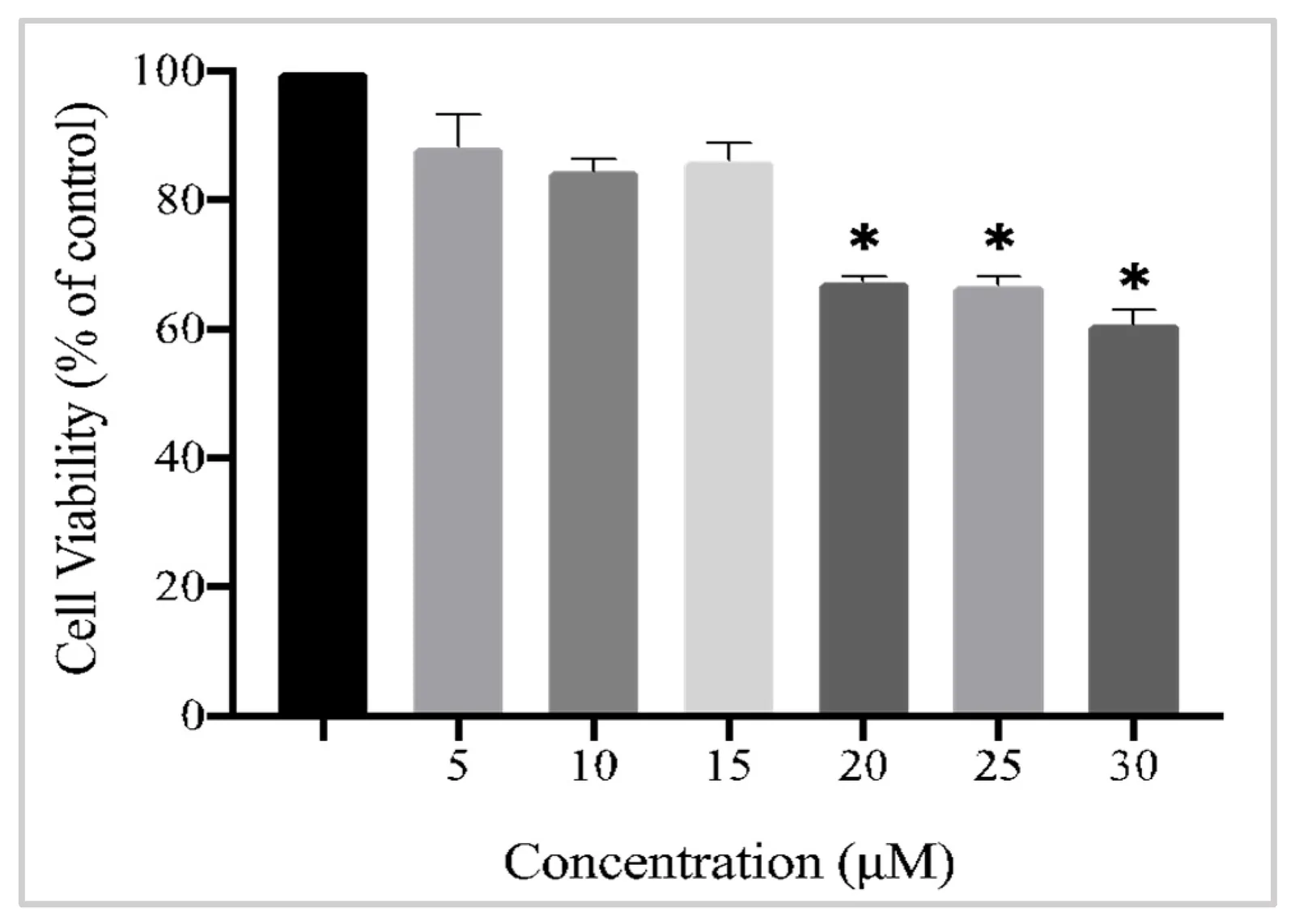
Effects of Aβ_42_-treated SH-SY5Y cell viability over a range of concentration (5 μM 30 μM) after 24 h. Data were expressed as mean ± SD from three independent experiments performed in triplicates. * *p* < 0.05, one-way ANOVA with Dunnett’s test vs. untreated group.

Based on our results, a concentration-dependent decrease in cell viability was noted upon 24 h incubation of SH-SY5Y cells with Aβ_42_ fibrils at 20 μM (67.44 ± 0.80), 25 μM (66.85 ± 1.37) and 30 μM (60.8 ± 2.30), respectively (Figure 3). Therefore, 30 μM was chosen as the optimal concentration to promote SH-SY5Y cell toxicity, as shown in the estimated 60% of cell survival following Aβ_42_ fibrillar treatment.

#### 3.1.3. Neuroprotective Effects of COR, ADE and DNPZ Against Aβ_42_-Induced Cytotoxicity

**Figure 4:**
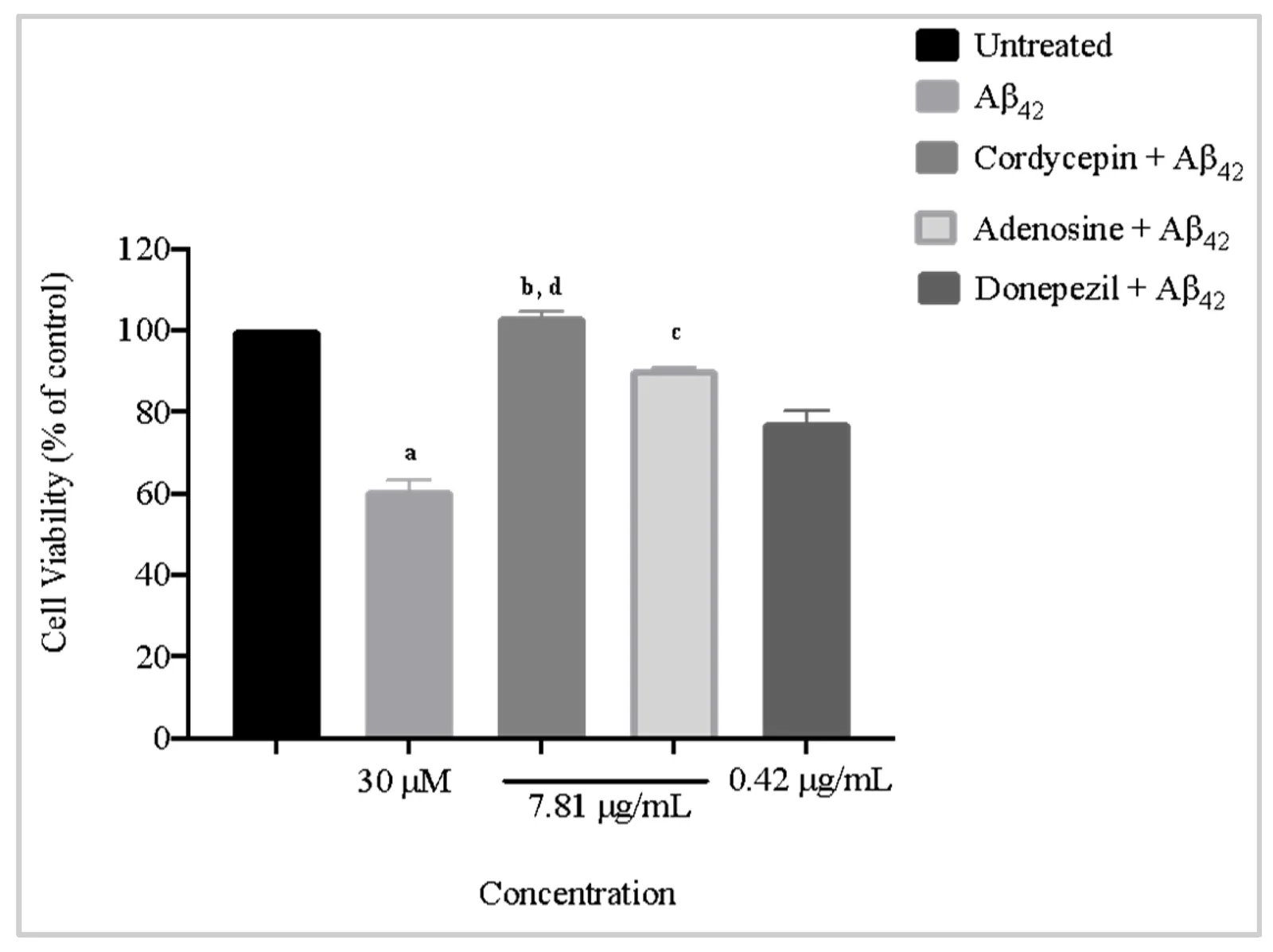
Effects of Aβ_42_, COR + Aβ_42_, ADE + Aβ_42_, and DNPZ + Aβ_42_ on SH-SY5Y cell viability after 24 h. The percentage of cell viability was calculated relative to the untreated group and presented as mean ± SD from three independent experiments performed in triplicate. ^a^ *p* < 0.01 vs. untreated cells; ^b^ *p* < 0.01 vs. Aβ_42_ treated group; ^c^ *p* < 0.05 vs. Aβ_42_ treated group; ^d^ *p* < 0.01 vs. DNPZ + Aβ_42_ treated group (one-way ANOVA with Tukey’s test).

As shown in Figure 4, SH-SY5Y cells treated with 30 μM Aβ_42_ fibrils had shown a pronounced increase in cytotoxicity (*p* < 0.01). Fortunately, the Aβ_42_-incubated cellular toxicity is effectively reduced upon pretreatment with COR and ADE. At 7.81 μg/mL, both pure compounds significantly restored SH-SY5Y cell viability to 103.22 ± 1.39 (*p* < 0.01, as compared to Aβ_42_ treated group) and 90.31 ± 0.65 (*p* < 0.05, as compared to Aβ_42_ treated group), respectively. In contrast, while pretreatment of DNPZ at 0.416 μg/mL does improve the cell viability from a visual perspective (77.31 ± 2.72), it nevertheless failed to report a significant outcome. Interestingly, higher cell viability is reported in cells pretreated with 7.81 μg/mL COR than that of 0.416 μg/mL DNPZ (*p* < 0.01). Our findings suggest that pretreatment with COR and ADE at 7.81 μg/mL protected SH-SY5Y neuroblastoma cells against Aβ_42_-induced cytotoxicity without inducing detectable cytotoxicity at the tested concentration. Collectively, these results support the potential neuroprotective effects of COR and ADE against Aβ_42_-induced cytotoxicity in this cellular model.

### 3.2. Prediction of Physicochemical and Pharmacokinetic Properties

#### 3.2.1. Drug-Likeliness Evaluation

The above treatment samples (COR, ADE and DNPZ) are subjected to drug-likeliness evaluation using Lipinski Rule of Five shown in Table 1. Log *P*, total polar surface area (TPSA), molecular weight (MW), number of hydrogen bond acceptors (nON) and donors (nOHNH) are among the fundamental descriptors known to represent drug absorption, solubility, and permeability. In this study, the Log *P* values of COR, ADE and DNPZ range from −0.8 to 4.2, the TPSA values from 38 to 140, and MW from 250 Da to 380 Da, respectively. Additionally, the nON and nOHNH values of all three ligands are reported to be within the range of 4–8 and 0–5, respectively. Compounds with fewer hydrogen-bonding functionalities, particularly hydrogen bond donors, may exhibit reduced polarity and greater hydrophobic character, which can favour interactions with hydrophobic regions of biological targets. This characteristic was particularly evident for DNPZ, which exhibited comparatively lower polarity and a higher Log *P* value than COR and ADE [49].

COR and ADE nevertheless satisfied the criteria of Lipinski Rule of Five by complying with ≥ three of these rules (Log *P* < 5, TPSA < 140 Å, MW < 500 Da, and nOHNH ≤ 10). Molecules coupled with optimum lipophilicity (Log *P* < 5) and lower TPSA values (TPSA < 140 Å) tend to exhibit higher lipid solubility which enhances membrane permeability and intestinal absorption, thereby contributing to a higher success rate of drug productivity [50,51]. Moreover, the smaller size and reduced bulkiness (MW < 500 Da) of both compounds further indicate that they can easily diffuse into the cells for absorption purposes [52]. Hence, COR and ADE are predicted to possess good oral bioavailability and favorable drug-like behavior [53,54,55].

#### 3.2.2. ADMET Filtering

The comparative ADMET (Absorption, Distribution, Metabolism, Excretion and Toxicity) profiles of COR, ADE and DNPZ are further calculated and depicted in Table 2. All three ligands are able to penetrate the Blood–Brain Barrier and be readily absorbed into the human intestinal tract for pharmacological effects to take place with respect to the positive BBB (Blood–Brain Barrier) and HIA (Human Intestinal Absorption) values. These results correspond to the estimated TPSA values above, supporting the highly permeable properties of COR and ADE. Various studies documented the essential role of COR in terminating polyadenylation and suppressing viral RNA synthesis [56,57], while ADE primarily acts as RNA precursors for transcription and nuclear signaling [58]. These critical biological processes usually occur in the nucleic-acid rich cellular compartment, which is in line with the predicted subcellular localization (nucleus).

P-glycoprotein (P-gp), along with cytochrome P450 superfamily are essential regulators of drug–drug interactions (DDI) that function to either limit or promote the bioavailability of orally administered drugs relying on their inducer/inhibitor status [59,60]. The foreign or toxic components within the drugs may undergo P-glycoprotein-mediated efflux for further liver metabolism (CYP450 enzymes) and renal clearance (ROCT) [61,62,63]. COR and ADE are portrayed as non-substrates and non-inhibitors of P-gp, various CYP450 enzymes and ROCT. These properties show that both compounds are less prone to initiating DDI via the P-gp or CYP450 pathways, minimizing the risk of toxic buildup in vulnerable populations that require intake of multiple medications.

Among the three ligands, only COR is reported positive for AMES toxicity test. The toxicity exerted by COR might have been related to its innate mechanism that interferes with polyadenylation, inhibiting RNA synthesis and ultimately exhibiting cytotoxicity. However, this process does not necessarily induce DNA mutations; hence, COR is not carcinogenic by AMES standards. To our knowledge, ADE is a fundamental building block for ATP, DNA and RNA synthesis, and is not involved in disruption of any cellular processes. Therefore, no toxicity or carcinogenicity is detected by the AMES toxicity test. Based on the LD_50_ results, both COR and ADE are considered relatively non-toxic in terms of acute toxicity settings, and higher doses are required to elicit death in 50% of the rat models. Overall, COR and ADE compounds are deemed suitable to be recognized as potential candidates in combating AD.

The ADMET analysis predicted that cordycepin and adenosine would have favorable pharmacokinetic properties. To further assess the predicted mutagenicity, cordycepin and adenosine were independently evaluated using ProTox-3.0. The predictions were consistent with the admetSAR results, whereby cordycepin was classified as mutagenic, whereas adenosine was predicted to be non-mutagenic. ProTox-3.0 additionally predicted both compounds to be non-carcinogenic, while only adenosine was classified as cytotoxic.

### 3.3. Network Pharmacology-Based Insights into Potential Molecular Mechanism

#### 3.3.1. Identification of Potential Therapeutic Targets

**Figure 5:**
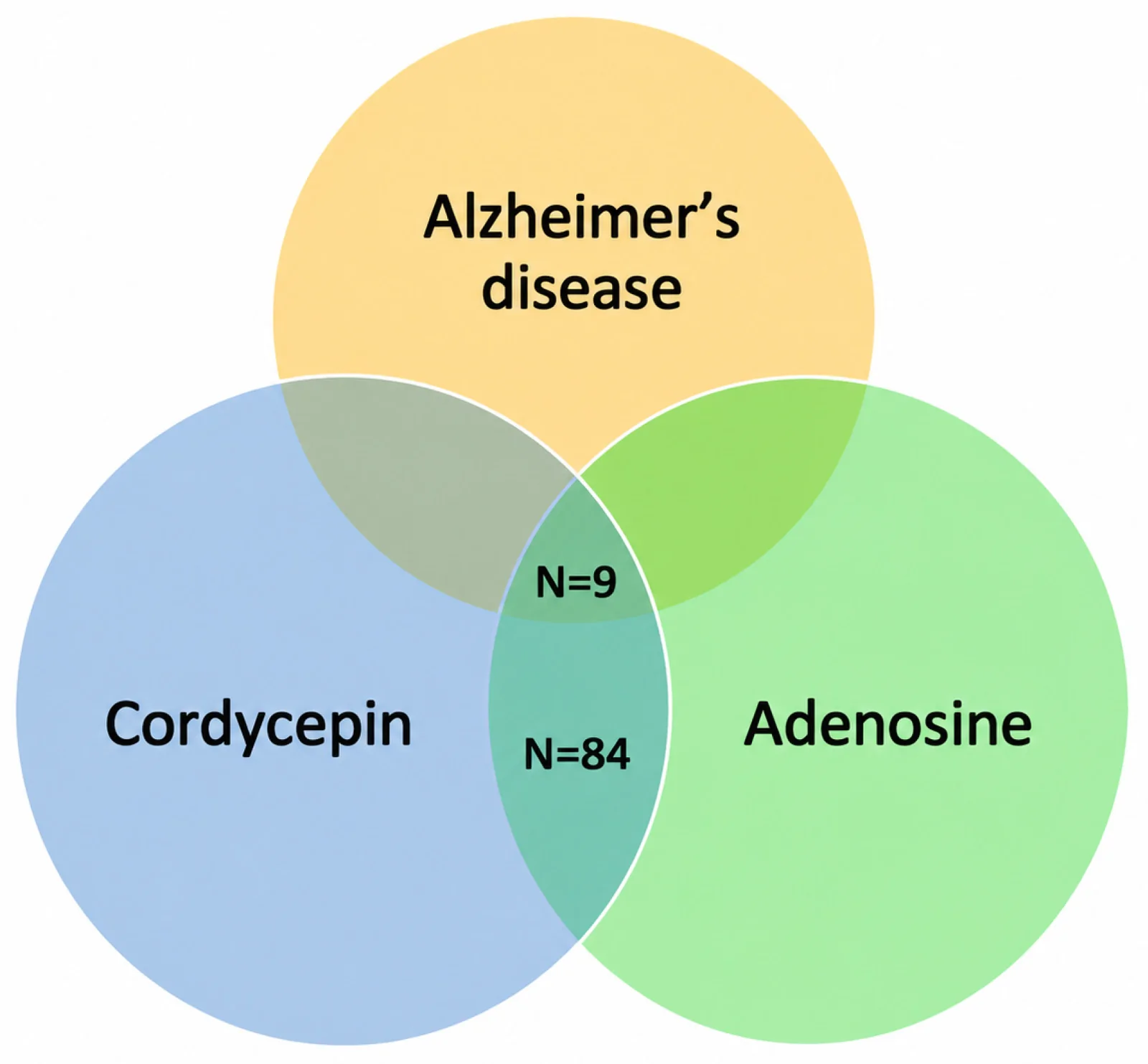
Identification of overlapping therapeutic targets. Venn diagram showing the overlap among AD-associated genes, COR targets, and ADE targets. AD-related genes were retrieved from GeneCards (https://www.genecards.org/; accessed 1 November 2025), whereas compound-associated targets were obtained from SwissTargetPrediction (http://www.swisstargetprediction.ch/; accessed 1 November 2025). A total of 84 shared targets were identified between COR and ADE. Among these, nine targets (*DNMT1*, *SLC6A3*, *SIGMAR1*, *AKT1*, *EHMT1*, *EZH2*, *FBP1*, *RPS6KA3*, and *NSD2*) overlapped with AD-associated genes and were designated as core AD-related targets for subsequent biological interpretation.

Potential therapeutic targets associated with the neuroprotective activity of cordycepin and adenosine against AD were identified through an intersection analysis of compound-related targets and AD-associated genes. A total of 96 putative targets were predicted for COR and 96 for ADE, while 500 AD-related genes were retrieved from GeneCards. To improve disease specificity and reduce background noise, only the top 500 protein-coding genes ranked by GeneCards relevance score were retained for downstream analyses. Venn analysis revealed 84 shared targets between COR and ADE, indicating substantial overlap in their predicted pharmacological profiles (Figure 5). These 84 shared targets were subsequently used for PPI, GO, and KEGG enrichment analyses to investigate their common biological functions. When intersected with AD-associated genes, nine core AD-related targets (*DNMT1*, *SLC6A3*, *SIGMAR1*, *AKT1*, *EHMT1*, *EZH2*, *FBP1*, *RPS6KA3*, and *NSD2*) were identified, suggesting that these genes may represent key mediators of the neuroprotective effects of both nucleosides.

Notably, several of the identified targets have previously been implicated in AD pathogenesis. *AKT1* and *RPS6KA3* participate in neuronal survival and synaptic signaling pathways, whereas *SIGMAR1* has been reported to exert neuroprotective functions through regulation of endoplasmic reticulum stress and mitochondrial homeostasis. The presence of epigenetic regulators including *DNMT1*, *EHMT1*, *EZH2* and *NSD2* further suggests that COR and ADE may influence AD through modulation of chromatin remodeling and gene expression. The substantial overlap in predicted targets between COR and ADE suggests that these structurally related nucleosides may share common pharmacological mechanisms. Nevertheless, differences observed in docking affinity and MD behavior indicate that each compound may preferentially modulate distinct signaling pathways, potentially contributing complementary neuroprotective effects.

#### 3.3.2. Protein–Protein Interaction Network and Hub Gene Analysis

**Figure 6:**
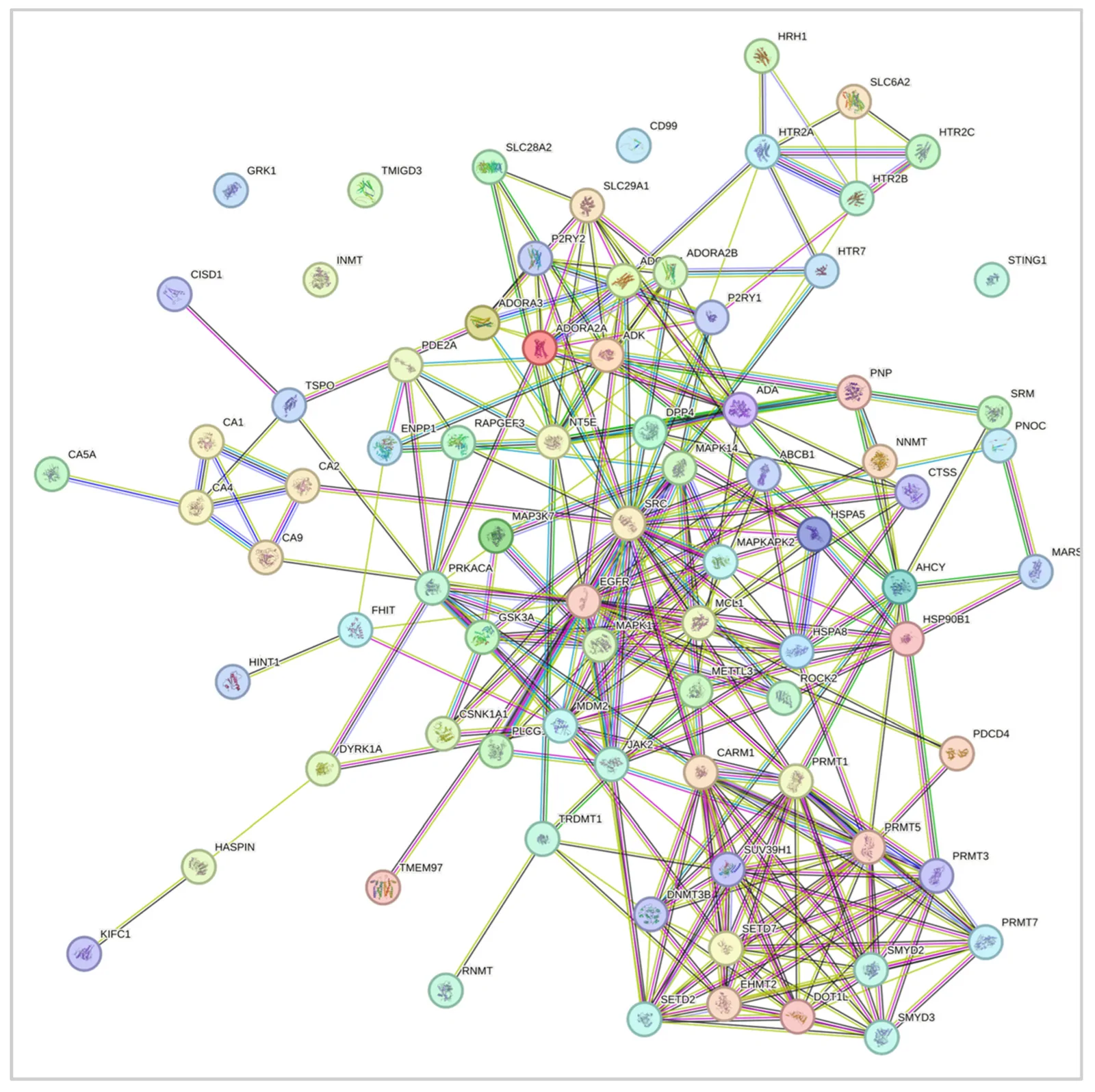
PPI network constructed from the 84 shared targets identified between COR and ADE. The network was constructed using the STRING database and visualized in Cytoscape 3.10.4. Nodes represent proteins and edges represent predicted or experimentally validated protein–protein interactions. Node size is proportional to degree centrality, whereby larger nodes indicate higher connectivity within the network. Highly connected proteins were identified as hub genes and considered potential key regulators of the neuroprotective activity of COR and ADE.

To further investigate the functional relationships among the 84 shared targets identified between COR and ADE, these targets were imported into the STRING database and visualized using Cytoscape. The resulting PPI network comprised 81 nodes and 293 edges, indicating extensive interactions among the candidate targets (Figure 6). Network topology analysis based on degree centrality identified *SRC*, *EGFR*, *PRMT5*, *MDM2*, *PRMT1*, *NT5E*, *CARM1*, *SUV39H1* and *MAPK1* as the principal hub genes, with SRC exhibiting the highest degree value (30), followed by EGFR (23). Additional highly connected nodes included *HSPA8*, *ADA*, *SETD7*, *SETD2*, *EHMT2*, *MCL1*, *PRMT3*, *ADK*, *DOT1L*, *JAK2* and *PRKACA.* Functional classification of the top 20 hub genes revealed four major biological themes: signal transduction, epigenetic regulation, purine metabolism and cellular stress responses (Table 3).

#### 3.3.3. KEGG and GO Functional Enrichment Analysis

**Figure 7:**
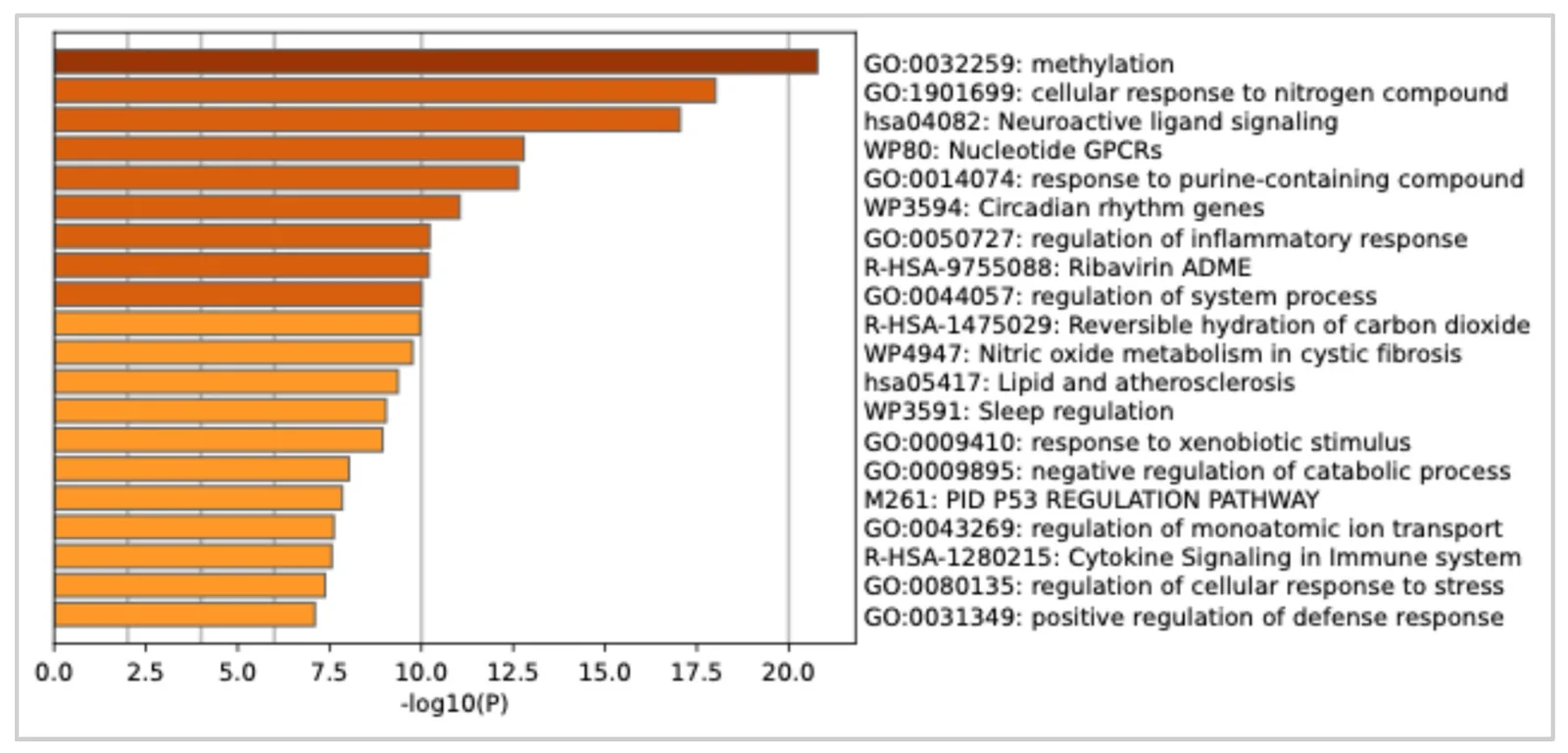
KEGG pathway enrichment analysis of the 84 overlapping targets shared between COR and ADE. Functional enrichment was performed using Metascape and categorized into Biological Process (BP), Molecular Function (MF) and Cellular Component (CC). The top enriched GO terms are displayed according to enrichment significance. The size of each node corresponds to the number of genes associated with the term, while color intensity reflects enrichment significance. GO analysis revealed significant enrichment in processes related to methylation, signal transduction, cellular response to stimuli and regulation of biological processes, suggesting potential roles of COR and ADE in neuronal signaling and epigenetic regulation.

KEGG (Figure 7) and GO (Supplementary Figure S2) enrichment analyses were performed using the 84 shared targets identified between COR and ADE to explore their biological significance. KEGG analysis demonstrated significant enrichment in neuroactive ligand–receptor interaction, calcium signaling pathway, inflammatory mediator regulation of TRP channels, Rap1 signaling pathway and chemokine signaling pathway. Seven additional pathways including metabolic pathways and lysine degradation were also enriched. The predominance of neuronal signaling and inflammatory pathways suggests that COR and ADE may exert neuroprotective effects through modulation of neurotransmission, intracellular calcium homeostasis and neuro-inflammatory responses.

### 3.4. Molecular Docking Analysis

Based on the network pharmacology findings together with the established roles of amyloidogenesis and lipid metabolism in AD, BACE1, LXRα and LXRβ were selected as representative targets for molecular docking and MD simulations.

#### 3.4.1. Ligand Selection

**Figure 8:**
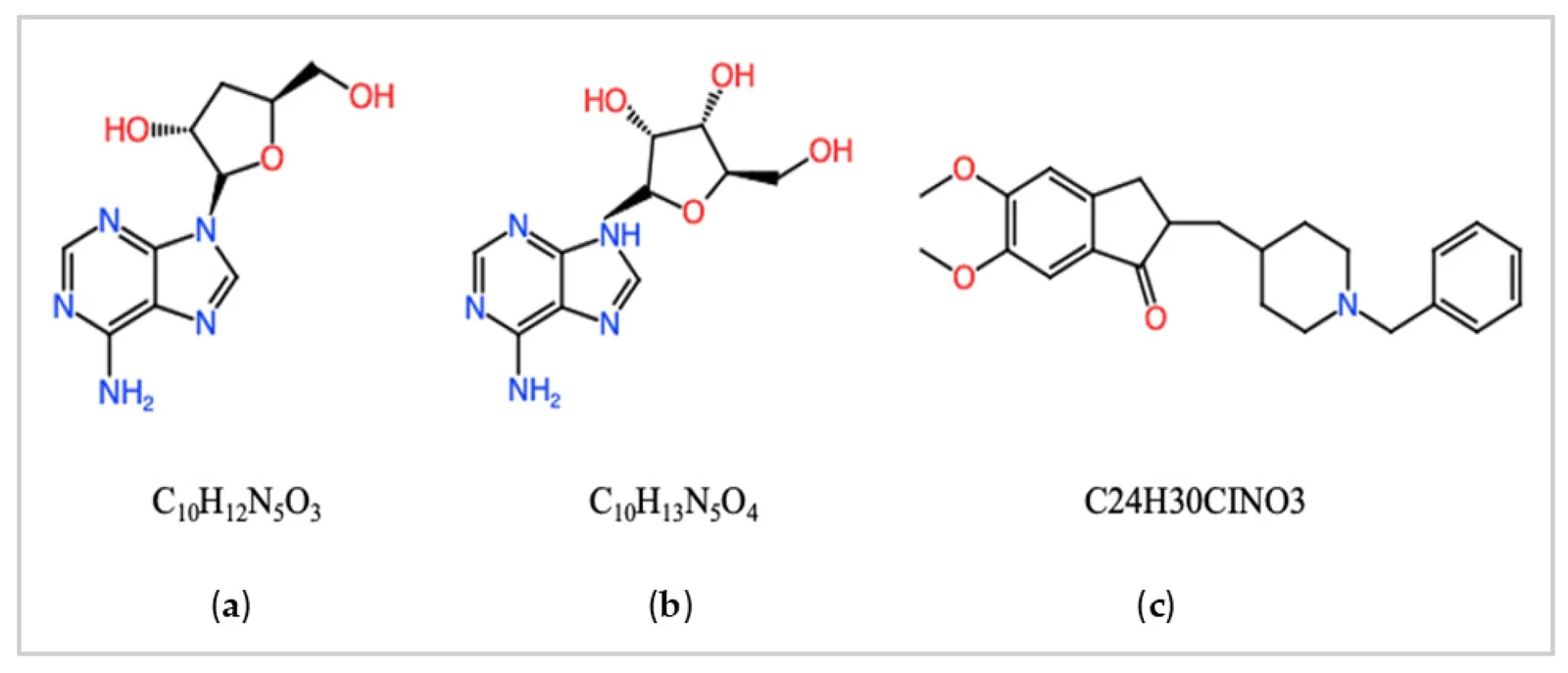
2D structural and molecular formula annotation of ligands; (**a**) COR, (**b**) ADE, and (**c**) DNPZ. In the chemical structures, oxygen (O) atoms are shown in red, nitrogen (N) atoms in blue, while carbon (C) atoms and the corresponding carbon skeleton are shown in black. Solid and dashed wedge bonds indicate the three-dimensional stereochemical configuration of the respective ligands.

Upon integrating the results obtained from drug-likeliness and ADMET screening, all three ligands were taken forward for molecular docking and MD simulation study. The 2D chemical structure and molecular formula of selected ligands are depicted in Figure 8.

#### 3.4.2. Ligand-Binding Site

**Figure 9:**
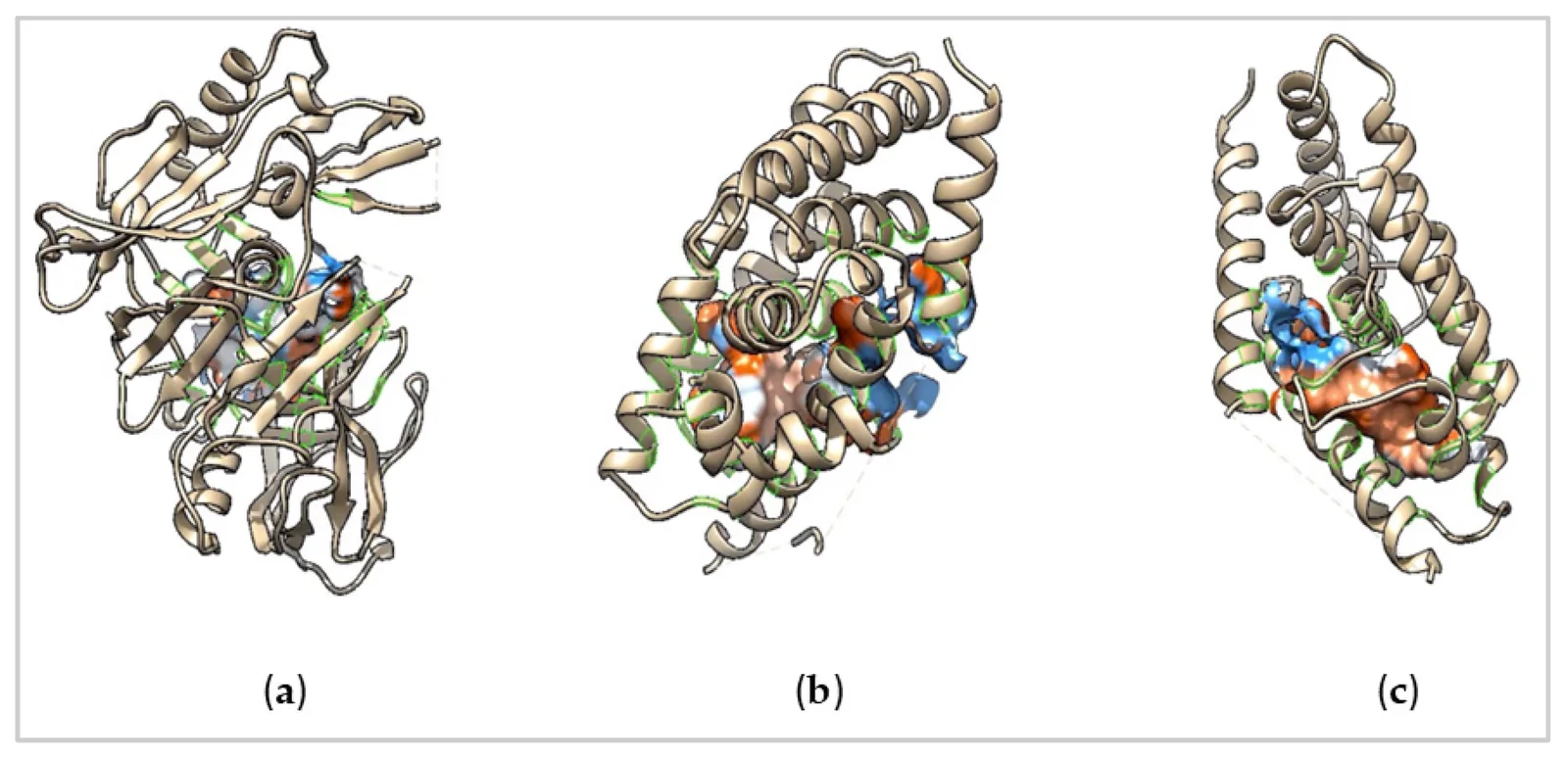
Geometry of the binding pocket in (**a**) BACE1, (**b**) LXR-α, and (**c**) LXR-β. The proteins are represented in cartoon form wherein the pockets indicated by multi-colored regions.

Prior to docking, identification of binding sites for ligand binding was performed through Castp. In this study, it is important to note that we did not designate the cuboidal AutoGrid4 box to envelop the entire receptor protein or just the co-crystallized ligand itself for docking analysis. Unlike previous publications, we opt to target the potential binding clefts or pockets located within a receptor protein that ligand molecules can attach to, commonly known as active sites. The identification of an active site is essential as it dictates the possible binding patterns of ligands for regulation of protein expression and biological activity. Based on Castp prediction, the putative ligand-binding pocket of each target protein was shown in Figure 9. The solvent-accessible surface area and volume for each binding pocket, as well as the amino acid residues contained within, are listed in Supplementary Table S1. A total of 39 amino acid residues lined the binding site of BACE1, while both LXR-α and LXR-β pockets are composed of 46 and 38 residues.

#### 3.4.3. Estimated Free Binding Energy Analysis

**Figure 10:**
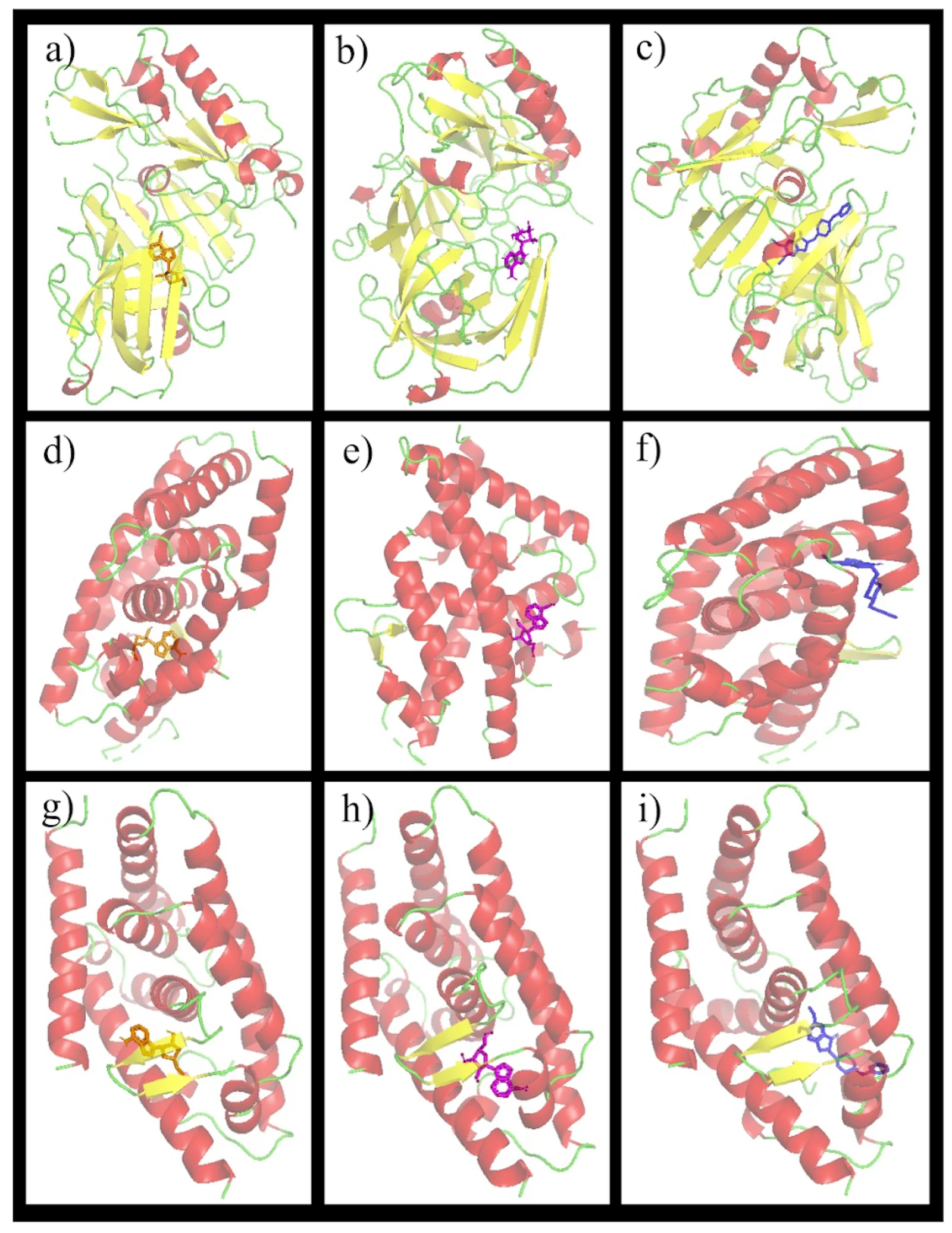
Docking conformation of selected ligands COR, ADE and DNPZ with (**a**–**c**) BACE1, (**d**–**f**) LXR-α, and (**g**–**i**) LXR-β, respectively. 3D structures of BACE1, LXR-α and LXR-β are shown in cartoon form, in which the helices, sheets and loops are collectively depicted in red, yellow and green colors. The ligands COR (orange), ADE (magenta) and DNPZ (blue) are represented in stick format.

Molecular docking of COR, ADE and DNPZ with AD-associated target proteins, namely BACE1 (6JSE), LXR-α (3IPQ) and LXR-β (6S5K) was carried out to identify the docking conformations and binding affinity. The docking conformations with the lowest binding energy of each ligand–protein complex are illustrated in Figure 10, whereas the estimated binding affinity are noted in Table 4. The key intermolecular interactions and binding residues identified for each ligand-protein complex are summarized in Supplementary Table S2.

Based on the binding energy analysis, DNPZ exerted the lowest binding energy when docked to BACE1 at −8.6 kcal/mol, followed by COR and ADE at −6.9 and −6.7 kcal/mol, respectively. This implies that DNPZ could bind favorably with BACE1 as compared to our compounds. Remarkably, both COR and DNPZ showed similar strong binding affinity of −6.8 kcal/mol against LXR-α. The higher binding energy of ADE at −5.9 kcal/mol, suggests that it might be a less potent LXR-α agonist. In contrast, COR demonstrated the highest binding preference against LXR-β (−7.8 kcal/mol), followed by ADE (−7.0 kcal/mol) and DNPZ (−6.5 kcal/mol). This indicates that COR seems to harbor a strong binding potential against the LXRs than that of ADE.

### 3.5. MD Simulations Analysis

#### 3.5.1. RMSD, RMSF, and Rg

**Figure 11:**
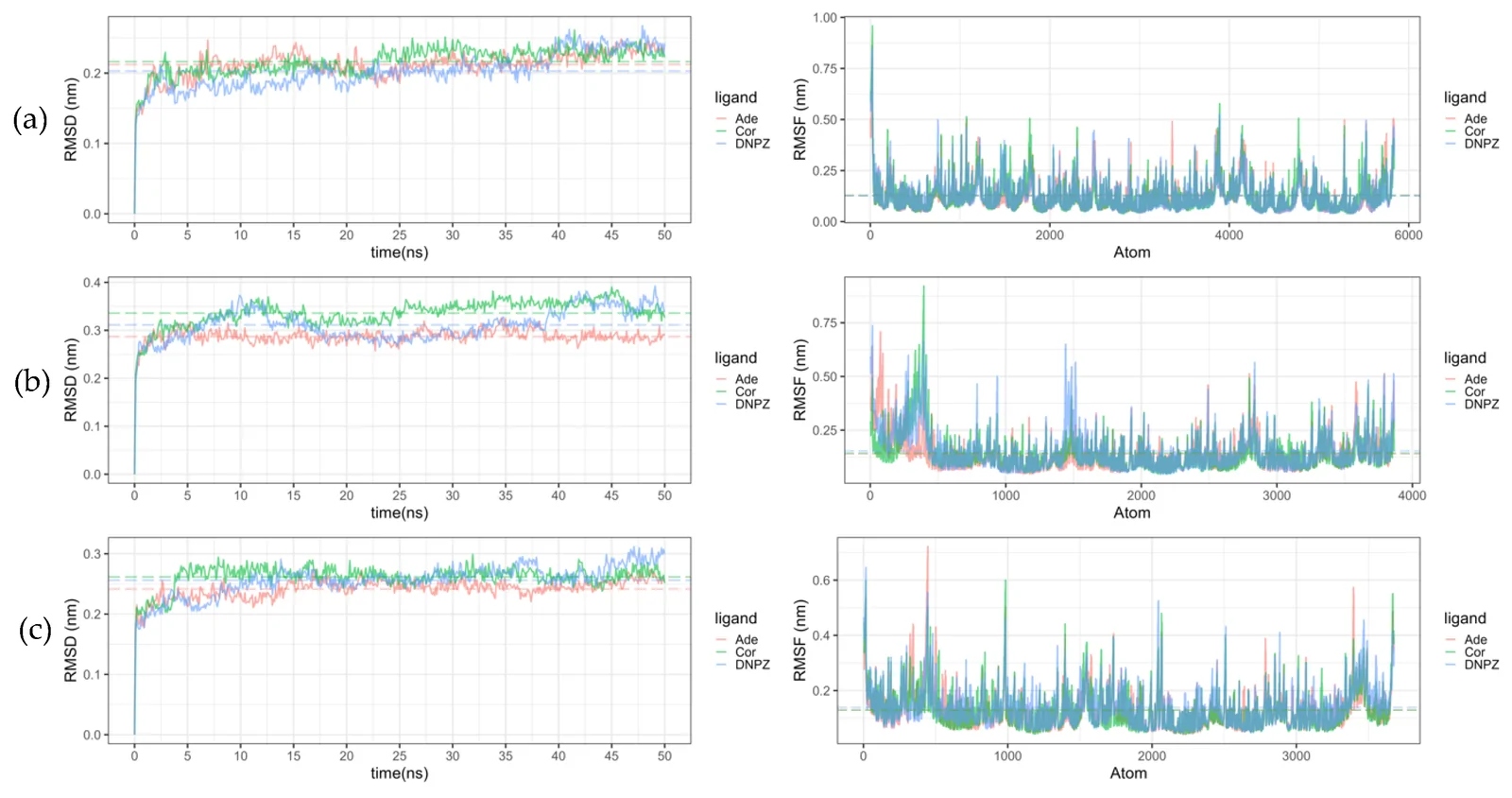
MD simulation analysis of protein–ligand complexes. Residual deviations and fluctuations are shown for complexes of COR, ADE, and DNPZ with (**a**) BACE1, (**b**) LXR-α, and (**c**) LXR-β, respectively, over a 50 ns MD simulation. The left panels of each subplot illustrate the RMSD of the docked complexes relative to the protein backbone, indicating the overall structural stability. The right panels display the RMSF of individual amino acid residues, reflecting their flexibility. The red, green, and blue lines consistently represent ADE, COR, and DNPZ complexes, respectively.

**Figure 12:**
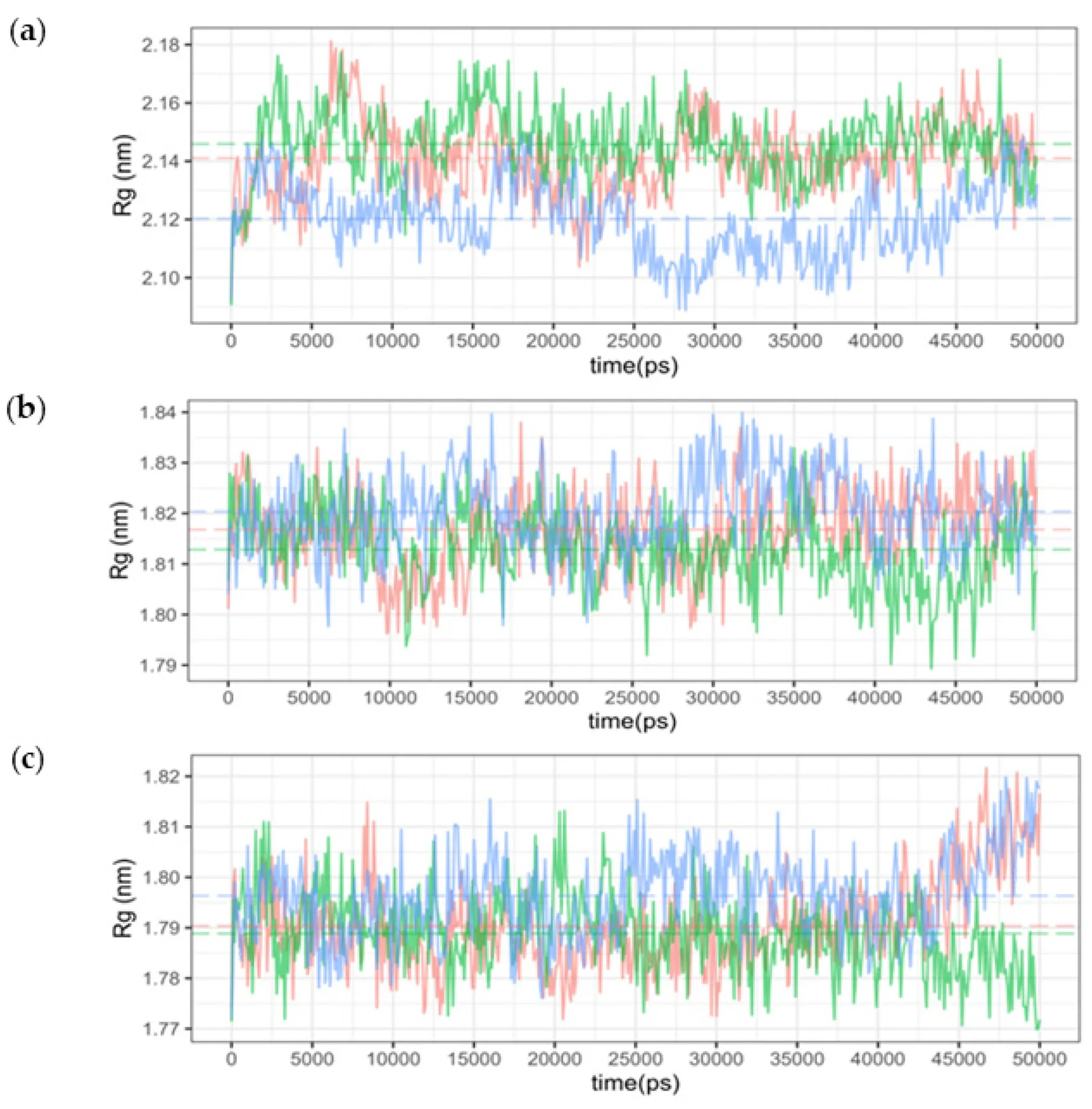
MD simulation analysis of protein–ligand complexes. The radius of gyration are shown for complexes of COR, ADE, and DNPZ with (**a**) BACE1, (**b**) LXR-α, and (**c**) LXR-β, respectively, over a 50 ns MD simulation. The red, green, and blue lines consistently represent ADE, COR, and DNPZ complexes, respectively.

The trajectories of COR, ADE and DNPZ in complex with BACE1, LXR-α and LXR-β acquired during MD simulations were measured using RMSD, RMSF and Rg analysis to better understand their respective dynamic behavior and stability profile (Figure 11 and Figure 12). RMSD offers insight on the global protein fluctuation while RMSF indicates residual fluctuation on the local level. High RMSD and RMSF values are known to reflect reduced structural stability and elevated mobility, respectively. On the other hand, Rg was applied to determine the global compactness of ligand–protein complexes. Low Rg values tend to indicate that the complex remains well-folded despite fluctuations that occur during the simulation period. In general, RMSD, RMSF and Rg fluctuation are interconnected as these parameters account for overall protein conformational stability and flexibility.

##### Trajectory Analysis of BACE1-Ligand Complexes

DNPZ demonstrated the most stable backbone conformation with an average RMSD of 0.20 ± 0.02 nm, consistent throughout the simulation, indicating minimal deviation from the initial structure. DNPZ portrayed relatively higher stability when bound to BACE1 compared to the other ligands. In contrast, COR and ADE exhibited slightly higher average RMSD values of 0.22 ± 0.02 nm and 0.21 ± 0.02 nm, respectively, with COR showing marginally increased fluctuation though still within a narrow range indicating overall stable binding. The average RMSF values were uniform across all complexes with a value of 0.13 ± 0.08 nm, reflecting limited local flexibility in protein residues during the binding of the different ligands. This stability in residue fluctuations implies a steady interaction between BACE1 and all ligands tested, with no major flexible regions disrupting binding. As for the average Rg, all complexes maintained similar compactness over time, ranging from 2.12 ± 0.01 nm for DNPZ to 2.15 ± 0.01 nm for COR. These narrow differences suggest the overall folding and structural integrity of the protein were well-preserved across all simulations, consistent with stable ligand engagement. Overall, the observed low RMSD and RMSF together with steady Rg values suggest that all three ligands form structurally stable complexes with BACE1 over the extended 50 ns simulation, with DNPZ showing a slight edge in dynamic stability.

##### Trajectory Analysis of LXR-α-Ligand Complexes

The backbone RMSD of all three complexes demonstrated overall stability throughout the simulations. COR showed a slightly higher average RMSD of 0.34 ± 0.03 nm, indicating marginally increased structural flexibility, although it remained within a stable range. In comparison, the ADE and DNPZ complexes exhibited very similar, stable RMSD values of 0.29 ± 0.02 nm and 0.31 ± 0.03 nm, respectively, suggesting minimal deviation from their initial structures. This stability in backbone dynamics points to a strong and consistent engagement of all three ligands. The average RMSF values were highly uniform across all complexes, ranging from approximately 0.14 ± 0.09 nm to 0.15 ± 0.10 nm. This limited local flexibility in the protein residues during ligand binding implies a steady and robust interaction without any major disruptions to the binding site. Furthermore, the average Rg values were nearly identical for all complexes, ranging from 1.81 ± 0.007 nm to 1.82 ± 0.008 nm. These narrow differences suggest that the overall folding and compactness of the protein were well-preserved across all simulations, consistent with stable ligand binding. Overall, the observed low RMSD and RMSF, combined with steady Rg values, collectively suggest that all three ligands form structurally stable complexes with LXR-α. While all are stable, the COR complex exhibits slightly more flexibility compared to the ADE and DNPZ complexes, which are remarkably similar in their dynamic stability.

##### Trajectory Analysis of LXR-β-Ligand Complexes

Similar to the LXR-α analysis, the LXR-β complexes also showed robust stability. The ADE complex displayed the most stable backbone conformation, with an average RMSD of 0.24 ± 0.02 nm. The COR and DNPZ complexes showed slightly higher RMSD values, at 0.26 ± 0.02 nm and 0.26 ± 0.03 nm respectively, though still indicating overall stable binding. The average RMSF values remained highly uniform across the LXR-β complexes, ranging from 0.13 ± 0.08 nm to 0.14 ± 0.08 nm, reflecting limited local flexibility and steady interactions. The consistent Rg values, which were approximately 1.79 ± 0.009 nm for all three complexes, confirm that the protein’s structural integrity and compactness were well-preserved. These results indicate that all three ligands form structurally stable complexes with LXR-β over the course of the simulation.

#### 3.5.2. Intermolecular Hydrogen Bond Occupancy Analysis

The formation of intermolecular hydrogen bond interactions is believed to induce a very significant effect on ligand–protein affinity and ensures the stability profiles of ligand–protein complexes by holding the conformational structure of protein pockets or active sites favorable for subsequent ligand–protein binding processes [64]. With this theory at hand, it is intuitively crucial to investigate the hydrogen bond network interactions of COR, ADE and DNPZ complexed to BACE1, LXR-α and LXR-β. The amino acid residues involved in hydrogen bonding and their respective occupancy throughout the 50 ns MD simulation were determined and compared with the hydrogen-bond interactions identified from molecular docking. A comparison of the intermolecular hydrogen-bond interactions identified from molecular docking and those observed during the MD simulations is provided in Supplementary Table S3. In Table 5, the presence of hydrogen bond donor–acceptor was computed according to the cut-off distance of ≤3.5 Å and cut-off angle (A-D-H) of 30° in VMD. Only the top three hydrogen bonds are shown for each complex.

The hydrogen bond occupancy analysis provided further insight into the dynamic stability and persistence of ligand–protein interactions throughout the 50 ns MD simulations. In the BACE1 complexes, all three ligands formed recurrent hydrogen bonds with active-site residues, although the occupancies were generally moderate, indicating transient yet stabilizing interactions. Notably, ARG152 emerged as a key interacting residue in the BACE1–COR and BACE1–DNPZ complexes, with occupancies of 17.73% and 18.73%, respectively, suggesting its involvement in ligand anchoring within the catalytic pocket. Similarly, interactions involving TRP100 and TYR222 were observed in the BACE1–COR and BACE1–ADE complexes, albeit with lower occupancies, reflecting fluctuating but repeated contact during the simulation.

In contrast, the LXRα–COR complex exhibited markedly higher hydrogen bond occupancies compared to other LXRα systems, particularly for COR:O2–THR302:OG1 (39.64%), COR:O3–HIS421:ND1 (25.50%), and COR:N5–SER264:OG (19.92%). These persistent interactions suggest a dominant role of hydrogen bonding in stabilizing cordycepin within the LXRα ligand-binding pocket. Conversely, COR and DNPZ formed fewer and weaker hydrogen bonds with LXRα, with all occupancies remaining below 10%, indicating that their binding stability is likely governed by alternative non-covalent interactions. A similar trend was observed for the LXRβ complexes. ADE demonstrated a highly persistent hydrogen bond with HIS435 (35.86%), accompanied by an additional interaction with TYR335 (17.13%), supporting its stable accommodation within the LXRβ binding site. COR also formed moderately stable hydrogen bonds with LXRβ, particularly involving GLU281, whereas DNPZ exhibited comparatively low hydrogen bond occupancies across all interactions. Collectively, these results indicate that while hydrogen bonding contributes to ligand stabilization, particularly for LXRα–COR and LXRβ–ADE complexes, overall binding stability during MD simulations is driven by a combination of persistent hydrogen bonds and complementary non-hydrogen-bond interactions, rather than hydrogen bond frequency alone.

### 3.6. MM/GBSA Analysis

#### 3.6.1. Binding Free Energy Calculation of BACE1-Ligand Complexes

To further quantify the binding stability of the protein–ligand complexes observed during MD simulations, MM/GBSA binding free energy calculations were performed on representative snapshots extracted from the final equilibrated trajectories. The total binding free energy (ΔG_bind) was decomposed into van der Waals (ΔE_VDW), electrostatic (ΔE_EL), polar solvation (ΔG_polar), and non-polar solvation (ΔG_nonpolar) components to elucidate the energetic contributions governing ligand binding. MM/GBSA analysis across BACE1, LXRα, and LXRβ complexes revealed clear target-dependent binding preferences among COR, ADE, and DNPZ, underscoring distinct energetic determinants governing ligand recognition by each receptor. In all systems, ΔG_TOTAL served as the primary metric for ligand ranking, with more negative values indicating stronger and more favorable interactions.

For BACE1 (Table 6), DNPZ emerged as the most potent binder (ΔG_TOTAL = −36.57 ± 3.18 kcal/mol), substantially outperforming COR and ADE. Energy decomposition showed that binding was predominantly van der Waals-driven, reflecting optimal hydrophobic packing and shape complementarity within the BACE1 active site. Although DNPZ incurred a higher polar solvation penalty, this unfavorable contribution was insufficient to offset its strong gas-phase stabilization, consistent with the hydrophobic nature of the BACE1 catalytic cleft. The relatively low standard deviation associated with the BACE1–DNPZ complex suggests a stable binding mode maintained throughout the simulation trajectory. In contrast, the higher variability observed for ADE, particularly in ΔG_TOTAL, indicates conformational instability or fluctuating interactions within the binding site, which may compromise its inhibitory potential.

These are indicative of DNPZ binding to BACE1 predominantly driven by hydrophobic and van der Waals interactions, with electrostatics playing a secondary role. This interaction profile aligns with established characteristics of potent BACE1 inhibitors, which often rely on optimal steric complementarity and hydrophobic engagement rather than strong electrostatic interactions alone. The comparatively weaker binding observed for COR and ADE suggests limited pocket accommodation and reduced interaction persistence, potentially translating to lower neuroprotective efficacy.

#### 3.6.2. Binding Free Energy Calculation of LXRα-Ligand Complexes

In contrast (Table 7), LXRα displayed a distinct selectivity profile, with COR exhibiting markedly stronger binding (ΔG_TOTAL = −31.18 ± 2.62 kcal/mol) relative to ADE and DNPZ, both of which showed weak and statistically unstable binding. The favorable interaction of COR with LXRα was primarily driven by a pronounced van der Waals contribution (E_VDW = −31.67 ± 2.69 kcal/mol), indicating superior steric complementarity and efficient hydrophobic packing within the LXRα ligand-binding domain. This strong gas-phase stabilization was accompanied by a relatively modest solvation penalty, allowing the net binding free energy to remain highly favorable and suggesting a well-balanced interaction between COR and the receptor environment.

By comparison, ADE and DNPZ exhibited substantially weaker gas-phase interaction energies (ΔG_GAS), reflecting suboptimal pocket occupancy and limited surface complementarity. The higher standard deviations associated with their total binding energies further indicate fluctuating binding modes and reduced interaction persistence over the simulation trajectory. Collectively, these energetic features suggest that COR adopts a more stable and well-defined binding pose within LXRα, whereas ADE and DNPZ fail to engage the hydrophobic core of the ligand-binding domain effectively, resulting in diminished affinity and stability. The comparatively larger standard deviations observed for ADE and DNPZ further suggest greater conformational flexibility and transient binding modes during the MD simulations, whereas the lower variance associated with COR is consistent with a more stable and persistent binding conformation throughout the trajectory.

#### 3.6.3. Binding Free Energy Calculation of LXRβ-Ligand Complexes

In Table 8, a contrasting trend was observed for LXRβ, where ADE demonstrated the strongest binding affinity (ΔG_TOTAL = −31.70 ± 2.13 kcal/mol), surpassing both COR and DNPZ. As with LXRα, this interaction was predominantly driven by van der Waals forces, indicating that ADE achieves optimal steric complementarity and hydrophobic engagement within the LXRβ ligand-binding pocket. The substantial gas-phase stabilization observed for the LXRβ–ADE complex reflects efficient pocket filling and persistent non-polar interactions that are maintained throughout the simulation.

ADE exhibited a favorable balance between intrinsic binding energy (ΔG_GAS) and solvation effects (ΔG_SOLV), resulting in a strongly negative total binding free energy with relatively low variance. This energetic balance suggests a stable and well-defined binding mode, with limited conformational fluctuation over time. In contrast, both COR and DNPZ showed weaker overall binding to LXRβ, accompanied by higher standard deviations in ΔG_TOTAL, indicative of less stable or more transient interactions. These findings imply that COR and DNPZ are less compatible with the structural and physicochemical features of the LXRβ ligand-binding domain, reinforcing the target-specific selectivity of ADE toward LXRβ.

Although molecular docking predicted favorable binding of COR toward LXRβ, MM/GBSA analysis identified ADE as the most stable binder following MD simulation. This difference reflects the distinct principles underlying the two approaches, as molecular docking evaluates ligand binding using a largely rigid receptor and a single binding pose, whereas MM/GBSA incorporates protein flexibility, solvent effects, and conformational sampling throughout the simulation trajectory. The larger standard deviations observed for the COR and DNPZ complexes further indicate greater conformational flexibility and transient binding modes, whereas the lower variance associated with ADE is consistent with a more stable and persistent interaction with the LXRβ binding pocket.

## 4. Discussion

Medicinal mushrooms are a valuable source of biologically active compounds that confer multiple pharmacological effects against different diseases, which include neurological-based ailments. In this study, we showed COR and ADE derived from *C. militaris* to confer neuroprotection against Aβ_42_-induced neurotoxicity on SH-SY5Y neuroblastoma cells. Drug-likeness and ADMET analyses demonstrated a good pharmacokinetic profile for both compounds with acceptable absorption and elimination properties. Toxicity prediction identified a possible mutagenicity signal for COR, but ADE was predicted to be non-mutagenic. Independent toxicity prediction was performed using ProTox-3.0 produced consistent mutagenicity predictions for both compounds. However, these findings represent computational predictions rather than experimental evidence and therefore require validation using established genotoxicity assays before definitive conclusions regarding safety can be drawn.

Several key targets associated with intracellular signal transduction, epigenetic regulation, purinergic metabolism and stress adaptation were identified from the PPI network, all of which have been implicated in AD pathogenesis. For example, SRC and EGFR regulate the PI3K/AKT and MAPK signaling cascades associated with amyloid-β toxicity, neuronal dysfunction, and AD progression. In contrast, NT5E, ADA, and ADK directly participate in adenosine metabolism and purinergic signaling, supporting the structural and functional relationship between COR and ADE. These findings suggest that the neuroprotective effects of COR and ADE are likely mediated through modulation of multiple interconnected pathways rather than a single molecular target.

KEGG and GO functional enrichment analysis further demonstrated that many of the overlapping gene modulators of COR and ADE are mainly involved in neuroactive ligand–receptor interaction, calcium signaling, and inflammatory responses. These pathways are responsible for governing synaptic plasticity and neurotransmission, which is consistent with previous studies portraying COR and ADE as neuromodulators capable of halting neurological-based disorders [65,66]. COR administration was found to inhibit production of pro-inflammatory mediators, thus preventing cerebral ischemia or reperfusion injury both in vitro and in vivo [67]. ADE similarly suppresses neuroinflammation and prolongs the neuronal survival period by mediating the cross talk between neurons and glial cells [68]. Sustained increase in ADE levels in the brain was found to positively correlate with better learning abilities [69].

Although BACE1, LXRα, and LXRβ were not identified among the overlapping disease-associated targets generated from the network pharmacology analysis, they were selected using a literature-guided approach because they are well-established therapeutic targets in Alzheimer’s disease with experimentally resolved crystal structures suitable for structure-based molecular modeling. The network pharmacology analysis primarily identified upstream regulators involved in signal transduction, chromatin regulation, neuronal survival, and purinergic signaling, suggesting that COR and ADE may exert pleiotropic effects that converge on these canonical AD pathways. Therefore, the molecular docking and molecular dynamics simulations should be regarded as literature-driven mechanistic validation rather than direct validation of the network-derived hub proteins.

To the best of our knowledge, this is the first study demonstrating the binding potential of COR and ADE isolated from *C. militaris* towards LXRs. Interestingly, DNPZ exhibited the strongest binding affinity towards BACE1, whereas COR and ADE preferentially interacted with LXRα and LXRβ, respectively. Molecular dynamics simulations further demonstrated that these interactions were predominantly driven by hydrophobic and dispersion forces, while electrostatic contributions remained comparatively small and were largely offset by polar solvation energies. This interaction pattern is characteristic of nuclear receptors and enzyme binding pockets with predominantly hydrophobic interiors, where ligand recognition depends largely on steric complementarity rather than strong electrostatic interactions. Notably, although molecular docking predicted favorable binding of COR towards LXRβ, MM/GBSA analysis following molecular dynamics simulation identified ADE as the more stable ligand. This difference reflects the distinct principles underlying the two approaches, as molecular docking evaluates ligand binding using a relatively rigid receptor and a single binding pose, whereas MM/GBSA incorporates protein flexibility, solvent effects, and conformational sampling throughout the simulation trajectory. Collectively, these findings provide a computational rationale for the selective modulation of BACE1 and LXR isoforms by COR and ADE, while recognizing that these predicted interactions require further experimental validation.

## 5. Limitation and Future Directions

While this study provides an integrated experimental and computational evaluation of the neuroprotective potential of COR and ADE, several limitations should be considered when interpreting the findings. The in vitro experiments were performed using SH-SY5Y neuroblastoma cells, a widely used model for investigating neurotoxicity and neuroprotection in Alzheimer’s disease. However, SH-SY5Y cells do not fully recapitulate the molecular and physiological characteristics of mature human neurons, including neuronal polarization, synaptic connectivity, and complex neuron–glia interactions. Therefore, the observed neuroprotective effects should be regarded as preliminary and warrant further validation in differentiated neuronal cultures, primary neurons, and appropriate in vivo models. Additionally, cell viability was assessed using the MTT assay, which primarily reflects cellular metabolic activity rather than direct cell survival or membrane integrity. Future studies incorporating complementary assays, such as lactate dehydrogenase (LDH) release, live/dead staining, or direct cell counting, would provide a more comprehensive evaluation of the neuroprotective effects of COR and ADE. Moreover, the presence of amyloid fibril was never directly assessed in the present study. In hindsight, it is recommended to include Thioflavin T fluorescence assay coupled with transmission electron microscope for better verification and visualization of amyloid fibrils formation.

The network pharmacology, ADMET prediction, molecular docking, and molecular dynamics approaches employed in this study provide complementary mechanistic insights into the potential actions of cordycepin and adenosine but do not constitute direct experimental validation. Accordingly, the predicted protein–ligand interactions, signaling pathways, and toxicity profiles, including the predicted mutagenicity of COR, should be regarded as computational hypotheses requiring confirmation through biochemical, molecular, and toxicological studies, such as target binding assays, gene expression analyses, functional pathway studies, and established genotoxicity assays (e.g., bacterial reverse mutation (Ames), micronucleus, or comet assays). Furthermore, the molecular dynamics simulations were performed over a 50 ns production trajectory using a single simulation for each protein–ligand complex. Although this simulation length was sufficient to evaluate the overall structural stability and binding behavior of the complexes, longer trajectories and independent replicate simulations would provide greater statistical confidence and may capture slower conformational transitions or infrequent binding events. Collectively, these future studies will further strengthen the mechanistic understanding and translational potential of COR and ADE for AD.

## 6. Conclusions

This study employed an integrated experimental and computational approach to investigate the neuroprotective potential of COR and ADE, two major bioactive nucleosides derived from *C. militaris*, against AD-related neurotoxicity. In vitro evaluation demonstrated that pretreatment with both compounds significantly attenuated Aβ_42_-induced neurotoxicity in SH-SY5Y neuroblastoma cells at non-toxic concentrations, supporting their neuroprotective effects in this cellular model. Network pharmacology analysis identified 84 shared targets between COR and ADE of which nine overlapped with AD-associated genes. Protein–protein interaction analysis further identified key hub genes involved in signal transduction, epigenetic regulation, and cellular stress responses, while GO and KEGG enrichment analyses highlighted pathways associated with neuroactive ligand–receptor interaction, calcium signaling, inflammatory regulation and methylation-related processes. These findings suggest that the neuroprotective effects of COR and ADE are mediated through coordinated regulation of multiple interconnected biological pathways rather than a single target.

Molecular docking and 50 ns MD simulations demonstrated favorable interactions of both compounds with BACE1, LXRα and LXRβ. Notably, COR exhibited the strongest binding characteristics towards LXRα, whereas ADE showed the strongest binding characteristics for LXRβ following molecular dynamics simulations and MM/GBSA analysis. These computational findings provide additional mechanistic insight into the potential modulation of amyloid processing, lipid metabolism, and neuronal signaling pathways, complementing the conventional amyloid-targeting strategy represented by BACE1. Overall, the combined experimental and computational findings support the potential of COR and ADE as bioactive nucleosides with multi-target neuroprotective properties. However, the predicted molecular interactions, signaling mechanisms, pharmacokinetic profiles, and toxicity assessments remain computational hypotheses and require further biochemical, pharmacological, toxicological, and in vivo validation before their neuroprotective potential for Alzheimer’s disease can be established.

## Figures and Tables

**Table 1:** Drug-likeliness evaluation of COR, ADE and DNPZ through Molinspiration.

Ligands	Lipinski Rule of Five				
	Log *P* ^1^	TPSA ^2^	MW ^3^	nOH ^4^	nOHNH ^5^
COR	−0.90	119.32	251.24	8	4
ADE	−0.85	139.55	267.24	9	5
DNPZ	4.10	38.78	379.50	4	0

^1^ Log *P*, logarithm of compound’s partition coefficient between *n*-octanol and water; ^2^ TPSA, total polar surface area; ^3^ MW, molecular weight; ^4^ nON, number of hydrogen bond acceptors; ^5^ nOHNH, number of hydrogen bond donors.

**Table 2:** Evaluation of ADMET profiles of COR, ADE and DNPZ through admetSAR.

Parameter	COR	ADE	DNPZ
Absorption			
HIA ^1^	0.9962	0.9227	1.0000
BBB ^2^	0.9743	0.9383	0.9943
P-gp ^3^ substrate	Non-substrate	Non-substrate	Substrate
P-gp inhibitor	Non-inhibitor	Non-inhibitor	Inhibitor
ROCT ^4^	Non-inhibitor	Non-inhibitor	Inhibitor
Subcellular distribution			
Subcellular localization	Nucleus	Nucleus	Mitochondria
CYP450 substrate			
CYP3A4	CYP3A4	CYP3A4	CYP3A4
CYP2C9	CYP2C9	CYP2C9	CYP2C9
CYP2D6	Non-substrate	Non-substrate	Substrate
CYP450 inhibitor			
CYP3A4	Non-inhibitor	Non-inhibitor	Non-inhibitor
CYP2C9	Non-inhibitor	Non-inhibitor	Non-inhibitor
CYP2C19	Non-inhibitor	Non-inhibitor	Non-inhibitor
CYP2D6	Non-inhibitor	Non-inhibitor	Inhibitor
CYP1A2	Non-inhibitor	Non-inhibitor	Inhibitor
Toxicity			
Carcinogenicity	Non-carcinogenic	Non-carcinogenic	Non-carcinogenic
AMES toxicity	Toxic	Non-toxic	Non-toxic
LD_50_ in rat (mol/kg)	1.9799	1.9715	2.9806
ProTox carcinogenicity	Inactive	Inactive	-
ProTox mutagenicity	Active	Inactive	-
ProTox cytotoxicity	Inactive	Active	-

^1^ HIA, Human Intestinal Absorption; ^2^ BBB, Blood–Brain Barrier; ^3^ P-gp, P-glycoprotein; ^4^ ROCT, Renal Organic Cation Transporter.

**Table 3:** Functional classification of the top hub genes identified from the protein–protein interaction network of the 84 shared targets shared between COR and ADE. Hub genes were selected based on degree centrality analysis performed in Cytoscape. The genes were subsequently grouped according to their predominant biological functions to facilitate interpretation of the molecular mechanisms potentially underlying the neuroprotective activities of COR and ADE.

Functional Cluster	Representative Hub Genes	Biological Relevance
Signal transduction	*SRC*, *EGFR*, *MAPK1*, *JAK2*, *PRKACA*	Regulation of neuronal survival and intracellular signaling
Epigenetic regulation	*PRMT1*, *PRMT3*, *PRMT5*, *CARM1*, *SUV39H1*, *SETD2*, *SETD7*, *EHMT2*, *DOT1L*	Chromatin remodeling and transcriptional regulation
Purine metabolism	*ADA*, *ADK*, *NT5E*	Adenosine metabolism and purinergic signaling
Stress response and cell survival	*MDM2*, *MCL1*, *HSPA8*	Cellular stress adaptation and apoptosis regulation

**Table 4:** Estimation of binding free energy of COR, ADE and DNPZ docked to BACE1, LXR-α and LXR-β.

Protein	Ligand	Binding Free Energy (kcal/mol)
BACE1	ADE	−6.7
	COR	−6.9
	DNPZ	−8.6
LXR-α	ADE	−5.9
	COR	−6.8
	DNPZ	−6.8
LXR-β	ADE	−7.0
	COR	−7.8
	DNPZ	−6.5

**Table 5:** The occupancy of hydrogen bonds in BACE1-ligand, LXR-α-ligand and LXR-β-ligand complexes.

Complex	Hydrogen Bond	Hydrogen Bond Occupancy
BACE1-COR	ARG152:NE-COR:O3	17.73%
	TRP100:NE1-COR:O2	16.14%
	COR:O3-PRO94:O	12.95%
BACE1-ADE	TYR222:OH-ADE:O3	15.14%
	ADE:O3-GLY58:O	11.95%
	TRP100:NE1-ADE:N3	5.98%
BACE1-DNPZ	ARG152:NE-DNPZ:O3	18.73%
	ARG152:NH1-DNPZ:O2	15.14%
	ARG152:NE-DNPZ:O2	3.59%
LXRα-COR	COR:O2-THR302:OG1	39.64%
	COR:O3-HIS421:ND1	25.50%
	COR:N5-SER264:OG	19.92%
LXRα-ADE	ADE:O4-HIS421:ND1	8.76%
	ADE:O4-SER418:O	2.79%
	ADE:N5-HIS383:ND1	2.39%
LXRα-DNPZ	GLN350:NE2-DNPZ:O2	2.39%
	ASN347:ND2-DNPZ:O3	1.39%
	SER310:OG-DNPZ:O2	1.00%
LXRβ-COR	LEU330:N-COR:O3	19.92%
	COR:O2-GLU281:OE2	18.92%
	COR:O2-GLU281:OE1	8.76%
LXRβ-ADE	ADE:N5-HIS435:ND1	35.86%
	ADE:O4-TYR335:OH	17.13%
	ADE696:N5-HIS383:ND1	2.39%
LXRβ-DNPZ	SER427:OG-DNPZ:O2	5.78%
	SER427:OG-DNPZ:O3	3.59%
	THR430:OG1-DNPZ:O3	1.39%

**Table 6:** MM/GBSA binding free energy decomposition of COR, ADE and DNPZ bound to BACE1, calculated from equilibrated MD trajectories; ΔG_bind comprises van der Waals, electrostatic, polar, and non-polar solvation contributions (kcal/mol).

Energy Contribution	Protein–Ligand Complex		
	BACE1-COR	BACE1-ADE	BACE1-DNPZ
E_VDW_	−18.06 ± 3.82	−14.64 ± 7.79	−37.23 ± 3.11
E_EL_	−4.76 ± 3.31	−2.08 ± 1.77	−6.31 ± 2.54
E_GB_	8.12 ± 2.15	5.26 ± 2.66	12.14 ± 1.78
E_SURF_	−2.78 ± 0.51	−2.26 ± 1.21	−5.18 ± 0.46
ΔG_GAS_	−22.82 ± 4.25	−16.71 ± 8.78	−43.53 ± 3.90
ΔG_SOLV_	5.34 ± 1.96	3.00 ± 1.62	6.96 ± 1.59
ΔG_TOTAL_	−17.48 ± 3.73	−13.71 ± 7.48	−36.57 ± 3.18

**Table 7:** MM/GBSA binding free energy components for COR, ADE and DNPZ in complex with LXRα, derived from equilibrated phases of 50 ns MD simulations and expressed in kcal/mol.

Energy Contribution	Protein–Ligand Complex		
	LXRα-COR	LXRα-ADE	LXRα-DNPZ
E_VDW_	−31.67 ± 2.69	−10.66 ± 8.89	−9.35 ± 11.30
E_EL_	−3.48 ± 1.72	−1.55 ± 1.76	−1.09 ± 2.09
E_GB_	8.57 ± 1.06	3.92 ± 3.12	2.37 ± 3.06
E_SURF_	−4.60 ± 0.22	−1.68 ± 1.40	−1.28 ± 1.53
ΔG_GAS_	−35.15 ± 2.89	−12.21 ± 10.18	−10.43 ± 12.87
ΔG_SOLV_	3.97 ± 0.96	2.24 ± 1.84	1.09 ± 1.79
ΔG_TOTAL_	−31.18 ± 2.62	−9.97 ± 8.55	−9.34 ± 11.52

**Table 8:** MM/GBSA binding free energy decomposition of COR, ADE and DNPZ bound to LXRβ, calculated from equilibrated MD trajectories and reported in kcal/mol.

Energy Contribution	Protein–Ligand Complex		
	LXRβ-COR	LXRβ-ADE	LXRβ-DNPZ
E_VDW_	−11.39 ± 11.46	−32.55 ± 2.20	−20.51 ± 11.31
E_EL_	−5.06 ± 5.65	−2.80 ± 0.92	−2.11 ± 2.02
E_GB_	6.82 ± 6.94	8.33 ± 0.68	5.16 ± 2.37
E_SURF_	−2.01 ± 2.02	−4.67 ± 0.19	−2.97 ± 1.60
ΔG_GAS_	−16.46 ± 16.53	−35.35 ± 2.29	−22.62 ± 11.34
ΔG_SOLV_	4.81 ± 5.01	3.65 ± 0.62	2.19 ± 1.80
ΔG_TOTAL_	−11.65 ± 11.86	−31.70 ± 2.13	−20.43 ± 11.27

## Data Availability

The data supporting the findings of this study are included in the published article and its Supplementary Materials. The computational data generated during the network pharmacology, molecular docking, ADMET/toxicity prediction, and molecular dynamics analyses are available from the corresponding author upon request.

## Supplementary Materials

The following supporting information can be downloaded at https://www.mdpi.com/article/10.3390/biomedicines14081862/s1, Figure S1: Validation of the docking grid box placement in relation to the CASTp-predicted binding pockets; Figure S2. Gene Ontology (GO) Enrichment Analysis of Molecular Function; Table S1: Solvent-accessible surface area, pocket volume, and amino acid residues comprising the predicted binding pockets of BACE1, LXR-α, and LXR-β; Table S2: Intermolecular interactions and key binding residues of COR, ADE, and DNPZ with BACE1, LXR-α, and LXR-β; Table S3: Comparison of molecular docking- and molecular dynamics-derived intermolecular hydrogen bond analyses for the BACE1–ligand, LXR-α–ligand, and LXR-β–ligand complexes.

## Abbreviations

The following abbreviations are used in this manuscript:

AD	Alzheimer’s disease
WHO	World Health Organization
Aβ	Amyloid-β
NFT	Neurofibrillary tangles
FAD	Familial AD
EOAD	Early-onset AD
LOAD	Late-onset AD
APP	Amyloid precursor protein
*PSEN1*	Presenilin 1
*PSEN2*	Presenilin 2
BACE1	β-secretase
sAPPβ	Soluble ectodomain fragment
C99	C-terminal fragment
AICD	APP intracellular domain
*APOE*	Apolipoprotein E
GWAS	Genome-wide association studies
RXRs	Retinoid X receptors
LXRs	Liver X receptors
ABCA1	ATP-binding cassette A1
*C. millitaris*	*Cordyceps millitaris*
HPLC	High-performance liquid chromatography
QTOF LC/MS-MS	Quadrupole time-of-flight liquid chromatography tandem mass spectrometry
NEAA	Non-essential amino acid
FBS	Fetal bovine serum
COR	Cordycepin
ADE	Adenosine
DNPZ	Donepezil
MTT	3-(4,5-dimethylhiazol-2-yl)-2,5-diphenyltetrazolium bromide
DMSO	Dimethyl sulfoxide
MNTC	Maximum non-toxic concentration
ADMET	Absorption, distribution, metabolism, excretion and toxicity
PDB	Protein data bank
Castp	Computed atlas of surface topography of proteins
MD	Molecular dynamics
CGenFF	CHARMM general force field
TIP3P	Transferable intermolecular potential with three-points
Na	Sodium
Cl	Chloride
PME	Particle mesh Ewald
LINCS	Linear constraint solver
RMSD	Root mean square deviation
RMSF	Root mean square fluctuation
Rg	Radius of gyration
VMD	Visual molecular dynamics
MM/GBSA	Molecular mechanics/generalized born surface area
ΔG_bind	Binding free energy
SD	Standard deviation
ANOVA	One-way analysis of variance
Log *P*	Logarithm of compound’s partition coefficient between *n*-octanol and water
TPSA	Total polar surface area
MW	Molecular weight
nON	Number of hydrogen bond acceptors
nOHNH	Number of hydrogen bond donors
HIA	Human intestinal absorption
BBB	Blood–brain barrier
Caco-2	Human colon adenocarcinoma
P-gp	P-glycoprotein
DDI	Drug–drug interactions
ROCT	Renal organic cation transporter
ΔE_VDW	Van der Waals
ΔE_EL	Electrostatic
ΔG_polar	Polar solvation
ΔG_nonpolar	Non-polar solvation
ΔG_GAS	Gas-phase interaction energies
ΔG_SOLV	Solvation effects
