# Supplementary material for: Multi-Target Neuroprotective Effects of Cordycepin and Adenosine from Cordyceps militaris Against Amyloid-β-Induced Neurotoxicity
Source: Biomedicines. 2026 Aug 20;14(8):1862. doi: 10.3390/biomedicines14081862 (PMC13509992; doi:10.3390/biomedicines14081862)
Supplement: Supplementary file 1 [file biomedicines-14-01862-s001.zip › Supplementary Figure S1.pdf]

Figure S1. Validation of the docking grid box placement in relation to the CASTp-predicted binding pockets

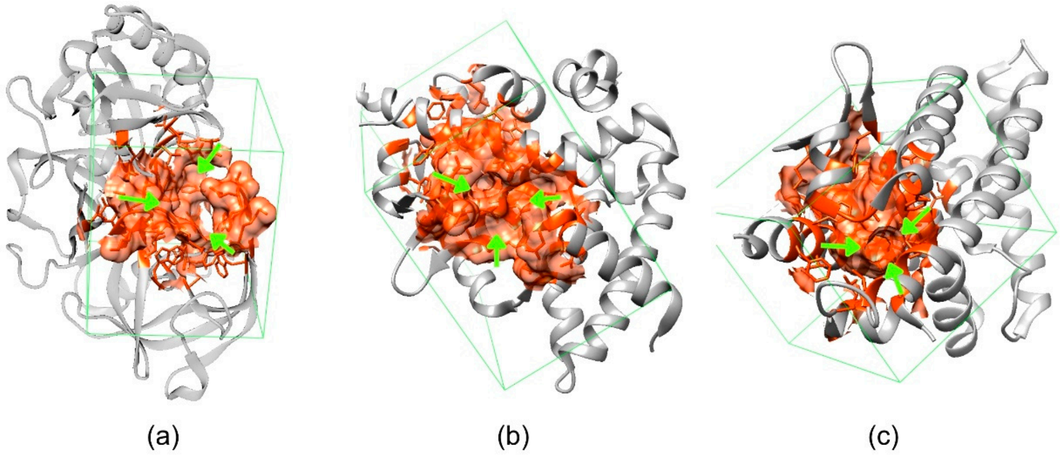

Supplementary Figure S1. Validation of the docking grid box placement in relation to the binding pockets predicted by CASTp. (a) BACE1, (b) LXRα and (c) LXRβ predicted binding pockets with representative views of the docking grid boxes. The residues of the binding pocket predicted by CASTp are in orange. The grid box of docking is in the form of a wireframe box in green. Green arrows indicate the entrance of each predicted binding pocket. The grid box was set to cover the entire binding cavity predicted by CASTp and all the residues of the pockets, thereby ensuring the inclusion of the full binding site during molecular docking.
