# Supplementary material for: Multi-Target Neuroprotective Effects of Cordycepin and Adenosine from Cordyceps militaris Against Amyloid-β-Induced Neurotoxicity
Source: Biomedicines. 2026 Aug 20;14(8):1862. doi: 10.3390/biomedicines14081862 (PMC13509992; doi:10.3390/biomedicines14081862)
Supplement: Supplementary file 1 [file biomedicines-14-01862-s001.zip › Supplementary Figure S2.pdf]

Figure S2. Gene Ontology (GO) Enrichment Analysis of Molecular Function

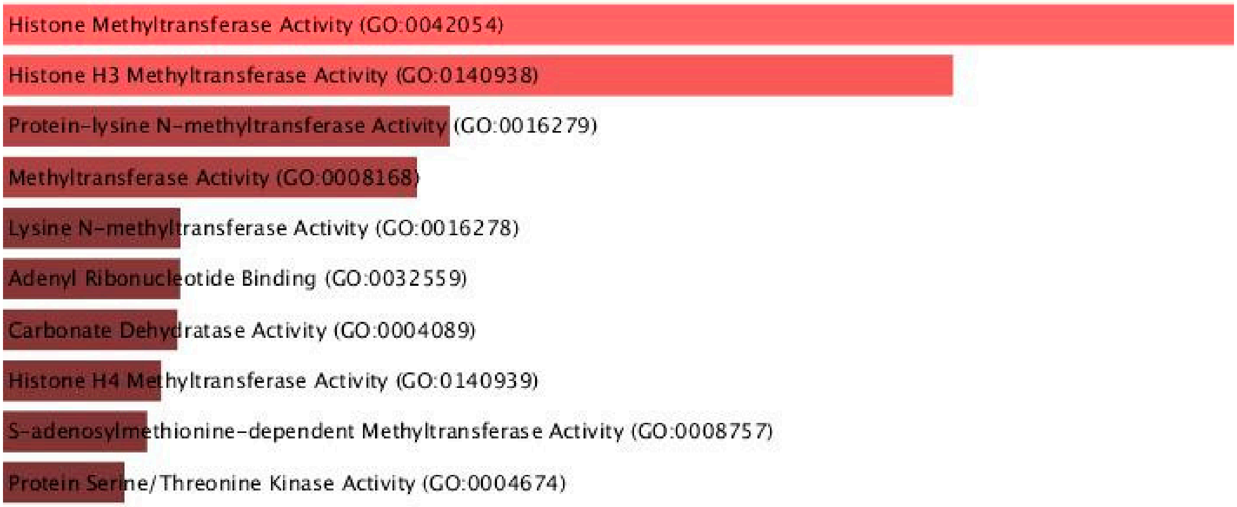

Supplementary Figure S2. Gene Ontology (GO) enrichment analysis of molecular function. The bar plot illustrates the significantly enriched Gene Ontology molecular function terms identified among the analysed targets. The enriched functions include histone methyltransferase activity (GO:0042054), histone H3 methyltransferase activity (GO:0140938), protein-lysine N-methyltransferase activity (GO:0016279), methyltransferase activity (GO:0008168), lysine N-methyltransferase activity (GO:0016278), adenyl ribonucleotide binding (GO:0032559), carbonate dehydratase activity (GO:0004089), histone H4 methyltransferase activity (GO:0140939), S-adenosylmethionine-dependent methyltransferase activity (GO:0008757), and protein serine/threonine kinase activity (GO:0004674).
