# Supplementary material for: Multi-Target Neuroprotective Effects of Cordycepin and Adenosine from Cordyceps militaris Against Amyloid-β-Induced Neurotoxicity
Source: Biomedicines. 2026 Aug 20;14(8):1862. doi: 10.3390/biomedicines14081862 (PMC13509992; doi:10.3390/biomedicines14081862)
Supplement: Supplementary file 1 [file biomedicines-14-01862-s001.zip › Supplementary Table S1.pdf]

Supplementary Table S1. Solvent-accessible surface area, pocket volume, and amino acid residues comprising the predicted binding pockets of BACE1, LXR-α, and LXR-β.

**Protein (PDB ID)**	**SA area**	**SA volume**	**Amino acid residues**
BACE1 (6JSE)	496.6	366.5	LYS33, SER34, GLY35, GLN36, GLY37, TYR38, LEU54, ASP56, GLY58, SER59, SER60, ASN61, ALA63, VAL93, TYR95, THR96, GLN97, TRP100, LYS131, PHE132, ILE134, TRP139, ILE142, ILE150, VAL194, TYR222, LYS248, ILE250, ASP252, SER253, GLY254, THR255, THR256, ARG259, ARG331, THR353, THR355, VAL356, ALA359
LXR-α (3IPQ)	609.3	403.4	VAL218, ALA220, GLN221, GLN 222, PHE254, PHE257, THR258, LEU260, ALA261, VAL263, SER264, GLU267, ILE268, PHE271, ILE295, VAL297, MET298, LEU299, GLU301, THR302, ARG304, ARG305, ILE313, THR314, PHE315, LEU316, LYS317, PHE326, ALA329, LEU331, PHE335, ILE336, ILE339, PHE340, ASP353, ALA357, ILE360, LEU417, VAL420, HIS421, GLN424, VAL425, LEU428, LEU435, LEU439, TRP443
LXR-β (6S5K)	392.5	187.0	GLN235, ASN239, PHE243, PHE268, PHE271, THR272, LEU274, ALA275, SER278, GLU281, ILE282, PHE285, ILE309, ILE311, MET312, LEU313, GLU315, THR316, ARG319, PHE329, LEU330, PHE340, ALA343, GLY344, LEU345, PHE349, ILE353, ILE374, HIS435, GLN438, VAL439, LEU442, LEU449, LEU453, TRP457

** SA, solvent accessible surface.*
