# Supplementary material for: Multi-Target Neuroprotective Effects of Cordycepin and Adenosine from Cordyceps militaris Against Amyloid-β-Induced Neurotoxicity
Source: Biomedicines. 2026 Aug 20;14(8):1862. doi: 10.3390/biomedicines14081862 (PMC13509992; doi:10.3390/biomedicines14081862)
Supplement: Supplementary file 1 [file biomedicines-14-01862-s001.zip › Supplementary Table S2.pdf]

**Supplementary Table S2.** Intermolecular interactions and key binding residues of COR, ADE, and DNPZ with BACE1, LXR-α, and LXR-β.

| Receptor protein | Docked ligands | Key amino acid residues | Type of interactions |
|---|---|---|---|
| BACE1 | COR | TRP100:NE1-COR:O<br>COR-TYR95<br>COR-TYR95<br>COR-ILE150 | H-Bond<br>HY (Pi-Pi Stacked)<br>HY (Pi-Pi Stacked)<br>Unfavorable linkage |
| | ADE | ADE:H-GLY58:O<br>ADE:H-GLN97:OE1<br>ADE-TYR95<br>ADE-TYR95<br>ADE:TRP400 | H-Bond<br>H-Bond<br>HY (Pi-Alkyl)<br>HY (Pi-Pi T-shaped)<br>Unfavorable linkage |
| | DNPZ | DNPZ:C-TYR222:OH<br>DNPZ:C-ASN61:OD1<br>DNPZ:C-ILE150:O<br>DNPZ:C-TYR95<br>DNPZ-TYR95<br>DNPZ-ILE134 | H-Bond (Carbon)<br>H-Bond (Carbon)<br>H-Bond (Carbon)<br>HY (Pi-Sigma)<br>HY (Pi-Pi Stacked)<br>HY (Pi-Alkyl) |
| LXR-α | COR | THR302:HG1-COR:N<br>COR:H-THR302:OG1<br>PH COR-PHE315<br>PHE315-COR<br>COR-LEU260<br>COR-ALA261<br>COR-MET298<br>COR-MET298 | H-Bond<br>H-Bond<br>HY (Pi-Pi Stacked)<br>HY (Pi-Pi Stacked)<br>HY (Pi-Alkyl)<br>HY (Pi-Alkyl)<br>HY (Pi-Alkyl)<br>HY (Pi-Alkyl) |
| | ADE | ADE:H-TRP443:O<br>ADE:H-THR292:O<br>SER418:HG-ADE:N<br>SER418:HG-ADE:O<br>HIS421:HD1-ADE:O<br>ADE-PRO370 | H-Bond<br>H-Bond<br>H-Bond<br>H-Bond<br>H-Bond<br>HY (Pi-Alkyl) |
| | DNPZ | ASN347:HD22-DNPZ:O<br>GLN350:HE21-DNPZ:O<br>DNPZ:C-ARG304:O<br>DNPZ:C-MET346:O<br>DNPZ:C-ASN347:O<br>THR314:CG2-DNPZ<br>DNPZ-LYS317 | H-Bond<br>H-Bond<br>H-Bond (Carbon)<br>H-Bond (Carbon)<br>H-Bond (Carbon)<br>HY (Pi-Sigma)<br>HY (Pi-Alkyl) |
| LXR-β | COR | COR:H-PHE329:O<br>THR316:OG1-COR:O<br>COR-PHE243<br>COR-PHE329<br>COR-PHE243<br>LEU274:C,O-COR<br>ALA275:N-COR | H-Bond<br>H-Bond<br>HY (Pi-Pi Stacked)<br>HY (Pi-Pi Stacked)<br>HY (Pi-Pi Stacked)<br>HY (Amide Pi-Pi Stacked)<br>Van der Waals |

| | | | |
|---|---|---|---|
| | ADE | THR316:OG1-ADE:O<br>ADE:ALA275<br>ADE:LEU345<br>ADE:TRP457<br>ADE:TRP457 | H-Bond<br>HY (Pi-Alkyl)<br>HY (Pi-Alkyl)<br>HY (Pi-Pi T-shaped)<br>HY (Pi-Pi T-shaped) |
| | DNPZ | SER432:HG-DNPZ:O<br>ALA306:CA-DNPZ:O<br>DNPZ-PHE440<br>DNPZ:C-ALA306<br>DNPZ:C-ILE309<br>VAL459-DNPZ<br>TRP457-DNPZ:C | H-Bond<br>H-Bond (Carbon)<br>HY (Pi-Pi Stacked)<br>HY (Alkyl)<br>HY (Alkyl)<br>HY (Alkyl)<br>HY (Pi-Alkyl) |

* COR, cordycepin; ADE, adenosine; DNPZ, donepezil; H-bond, hydrogen bond; HY, hydrophobic contacts.
