# Supplementary material for: Multi-Target Neuroprotective Effects of Cordycepin and Adenosine from Cordyceps militaris Against Amyloid-β-Induced Neurotoxicity
Source: Biomedicines. 2026 Aug 20;14(8):1862. doi: 10.3390/biomedicines14081862 (PMC13509992; doi:10.3390/biomedicines14081862)
Supplement: Supplementary file 1 [file biomedicines-14-01862-s001.zip › Supplementary Table S3.pdf]

Supplementary Table S3. Comparison of molecular docking- and molecular dynamics-derived intermolecular hydrogen bond analyses for the BACE1–ligand, LXR-α–ligand, and LXR-β–ligand complexes.

Receptor protein	Ligand	Amino acid residues involved in hydrogen bonding	Molecular docking	MD simulations
BACE1	COR	ARG152:NE		✓
		TRP100:NE1	✓	✓
		ARG152:NH1		✓
		GLY58:O		✓
	ADE	GLY58:O	✓	
		PRO94:O		✓
		GLN97:OE1	✓	
		TRP100:NE1		✓
	DNPZ	ASN61:OD1	✓	
		ILE150:O	✓	
		ARG152:NE		✓
		TYR222:OH	✓	
LXR-α	COR	THR302:HG1	✓	
		THR302:OG1	✓	
		PHE257:O		✓
		ALA220:O		✓
	ADE	TRP443:O	✓	
		THR292:O	✓	
		SER418:HG	✓	
		HIS421:HD1	✓	
		HIS421:ND1		✓
	DNPZ	MET346:O	✓	
		ARG304:O	✓	
		ASN347:O	✓	
		GLN350:HE21	✓	
		ASN347:HD21		✓
		ASN347:HD22	✓	
LXR-β	COR	THR316:OG1	✓	✓
		PHE329:O	✓	✓
	ADE	THR316:OG1	✓	
	DNPZ	ALA306:CA	✓	
		SER432:HG	✓	
